# Cost-effective circadian mechanism: rhythmic degradation of circadian proteins spontaneously emerges without rhythmic post-translational regulation

**DOI:** 10.1016/j.isci.2021.102726

**Published:** 2021-06-14

**Authors:** Roktaek Lim, Junghun Chae, David E. Somers, Cheol-Min Ghim, Pan-Jun Kim

**Affiliations:** 1 Department of Biology, Hong Kong Baptist University, Kowloon, Hong Kong; 2 Department of Physics, Ulsan National Institute of Science and Technology, Ulsan 44919, Republic of Korea; 3 Department of Molecular Genetics, The Ohio State University, Columbus, OH 43210, USA; 4 Center for Applied Plant Sciences, The Ohio State University, Columbus, OH 43210, USA; 5 Center for RNA Biology, The Ohio State University, Columbus, OH 43210, USA; 6 Arabidopsis Biological Resource Center, The Ohio State University, Columbus, OH 43210, USA; 7 Department of Biomedical Engineering, Ulsan National Institute of Science and Technology, Ulsan 44919, Republic of Korea; 8 Center for Quantitative Systems Biology & Institute of Computational and Theoretical Studies, Hong Kong Baptist University, Kowloon, Hong Kong; 9 State Key Laboratory of Environmental and Biological Analysis, Hong Kong Baptist University, Kowloon, Hong Kong; 10 Abdus Salam International Centre for Theoretical Physics, 34151 Trieste, Italy

**Keywords:** Mathematical biosciences, Systems biology, In silico biology

## Abstract

Circadian protein oscillations are maintained by the lifelong repetition of protein production and degradation in daily balance. It comes at the cost of ever-replayed, futile protein synthesis each day. This biosynthetic cost with a given oscillatory protein profile is relievable by a rhythmic, not constant, degradation rate that selectively peaks at the right time of day but remains low elsewhere, saving much of the gross protein loss and of the replenishing protein synthesis. Here, our mathematical modeling reveals that the rhythmic degradation rate of proteins with circadian production spontaneously emerges under steady and limited activity of proteolytic mediators and does not necessarily require rhythmic post-translational regulation of previous focus. Additional (yet steady) post-translational modifications in a proteolytic pathway can further facilitate the degradation's rhythmicity in favor of the biosynthetic cost saving. Our work is supported by animal and plant circadian data, offering a generic mechanism for potentially widespread, time-dependent protein turnover.

## Highlights

• Rhythmic degradation of circadian proteins lowers the cost of protein synthesis
• This rhythmic degradation emerges without rhythmic post-translational regulation
• Extra, yet steady post-translational modifications enhance degradation rhythmicity
• This mechanism hints at how organisms afford the price of daily biological rhythms

## Graphical abstract

**Figure:**
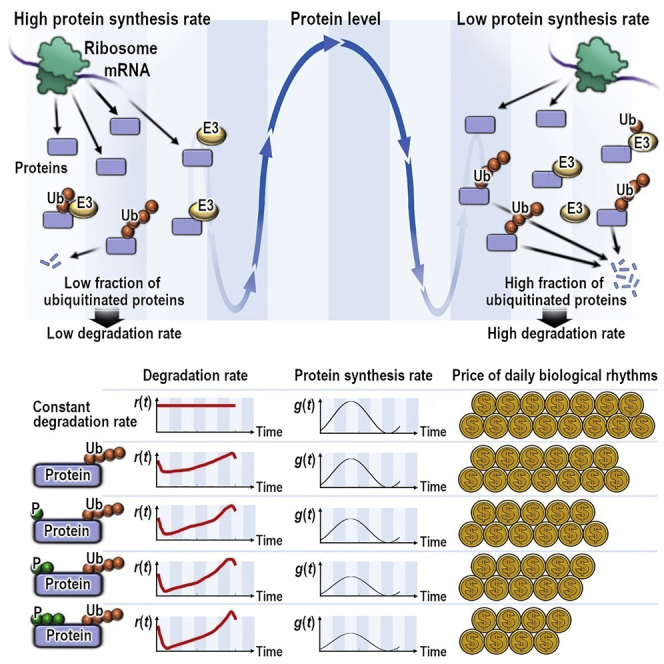

## Introduction

Circadian clocks in various organisms generate endogenous molecular oscillations with ∼24-h periodicity, enabling physiological adaptation to diurnal environmental changes caused by the Earth's rotation around its axis. Circadian clocks play a pivotal role in maintaining biological homeostasis, and the disruption of their function is associated with a wide range of pathophysiological conditions (Brody and Harris, 1973; Gachon et al., 2004; Nagel and Kay, 2012; Sehgal et al., 1994). Despite the ongoing efforts to understand the mechanisms of circadian clocks and their downstream regulation, almost absent in the predominating perception is *the price of daily biological rhythms*, a key concept that we will delineate below.

**Figure 1:**
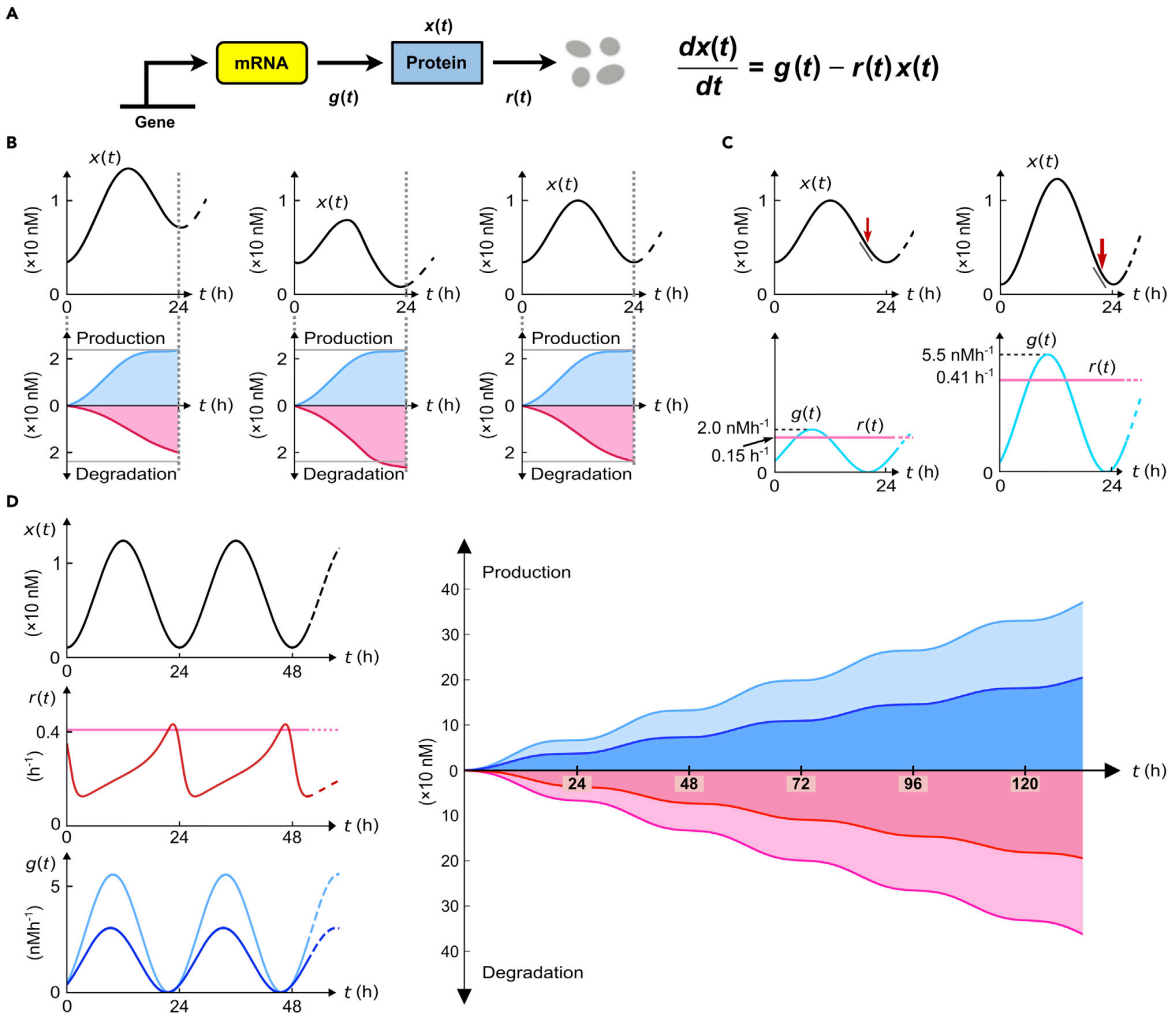
Relationship between circadian protein degradation and biosynthetic costs (A) Schematic diagram of protein production and degradation. x(t), g(t), and r(t) represent protein concentration, synthesis rate, and degradation rate over time t, respectively. (B) Each upper panel shows protein concentration profile x(t), and the lower panel shows the corresponding total protein amount produced (bluish, $\overset{t}{\underset{0}{\int }}g\left(t^{\prime }\right)dt^{\prime }$) or degraded (reddish, $\overset{t}{\underset{0}{\int }}r\left(t^{\prime }\right)x\left(t^{\prime }\right)dt^{\prime }$) over time t from t=0 h. Gray horizontal lines in each lower panel indicate the protein amount produced until t=24 h in order to guide visual comparison with the degraded protein amount. (C) The upper panels show different waveforms of x(t) (a sharper waveform on the right-hand side), and the lower panels show the corresponding g(t) and r(t) when r(t) is constant over time. In each lower panel, r(t) takes a value close to the theoretical minimum (${\text{max}}_{t}\left[-x^{\prime }\left(t\right)/x\left(t\right)\right]$) determined at the time point with an arrow in the upper panel (for details, see STAR Methods, relationship between protein abundance profiles and biosynthetic costs). A sloped segment in each upper panel indicates the rapidness of protein degradation required at the time point with the arrow. (D) Given the protein profile x(t) in (C), the left panels exhibit r(t) when r(t) is constant or rhythmic (darker color than constant r(t)). They also show the corresponding g(t) (light and dark blue for the cases of constant and rhythmic r(t), respectively). The right panel shows the total protein amount produced (bluish) or degraded (reddish) over time, plotted in a similar fashion to (B). Darker (lighter) colors in the right panel correspond to the case of the rhythmic (constant) r(t) in the left panel. Given the protein profile, the rhythmic degradation rate is associated with a less amount of protein production, i.e., a lower proteosynthetic cost than the constant degradation rate (STAR Methods, relationship between protein abundance profiles and biosynthetic costs).

Because protein abundances controlled by the circadian system and/or diurnal environmental changes are periodic over time, the proteins must be degraded by the same amount as a total of their synthesis, in each period of the oscillations; otherwise, the proteins would be either accumulated or depleted over the periods without precise repetition of their abundances (Figures 1A and 1B and STAR Methods, relationship between protein abundance profiles and biosynthetic costs) (Jo et al., 2018). This counterbalance between protein synthesis and degradation back to the baseline every period could only be sustained at the expense of ever-replayed protein synthesis on a daily basis—a circadian version of the Red Queen's race (Carroll, 1872). We previously demonstrated that these daily rounds might be particularly a burden to the case of highly oscillatory proteins with sharp waveforms, because establishing these waveforms requires rapid protein degradation at their falling phases (even involving the examples of sub-hour-long protein half-lives) and thereby substantial proteosynthesis for their replenishment (Figure 1C and STAR Methods, relationship between protein abundance profiles and biosynthetic costs) (Jo et al., 2018). In this context, our aforementioned notion, the price of daily biological rhythms, refers to the inevitable expense of protein synthesis in maintaining lifelong circadian rhythms of a given organism. Although not at the whole organism level, our rough estimation suggests that ∼4% of mouse liver proteins with circadian or diurnal rhythmicity account for ∼20% of the total mouse liver protein synthesis, under the assumption of the constancy of protein half-lives over time (STAR Methods, biosynthetic cost of mouse liver proteins in the case of constant protein half-lives). As mathematically proven in our previous study (Jo et al., 2018), constant half-life indicates a constantly short-lived protein, which is degraded all the time at least as rapidly as required at the falling phase of the oscillation (STAR Methods, relationship between protein abundance profiles and biosynthetic costs). This constantly high protein loss imposes a severe proteosynthetic load (Figure 1C and STAR Methods, relationship between protein abundance profiles and biosynthetic costs). One of the mechanisms to relieve such a biosynthetic burden is that proteins with oscillating abundances are rhythmically degraded (Jo et al., 2018; Kiba et al., 2007; Lück et al., 2014; van Ooijen et al., 2011), rather than only rhythmically produced as commonly assumed with circadian mRNA expression or translation rates. Specifically, a degradation rate that selectively peaks at the falling phase of an abundance oscillation, but stays relatively low elsewhere, can reduce a gross protein loss and save much of the proteosynthesis in return (Jo et al., 2018). Figure 1D illustrates such a drop of 45% of proteosynthesis, and a similar or even more saving was anticipated at least for some plant and mammalian core clock proteins (Jo et al., 2018).

This observation of rhythmic protein degradation as one plausible solution to the price of daily biological rhythms raises a fundamental question: what is the underpinning molecular mechanism of such rhythmic degradation? Predominant studies on the rhythmic protein stability have been focusing on the presence of particular post-translational, proteolytic pathways, presumably under active circadian or diurnal control, such as the pathways with time-of-day-specific kinase, F-box protein, and autophagic activities (Cha et al., 2017; Kiba et al., 2007; Lamia et al., 2009; Toledo et al., 2018). However, it is unclear whether the repertoire of such oscillatory, proteolysis-related processes is really diverse enough to cover all the substrates in our question. Moreover, their contribution to the biosynthetic cost reduction can be rather limited, if the proteolytic mediators themselves have the own oscillating abundances that incur the extra biosynthetic costs.

In this study, we suggest that the rhythmic degradation of proteins with daily oscillatory production does not necessarily require the rhythmic post-translational regulation under circadian or diurnal control. Rather, steady proteolytic mediators suffice to induce clearly rhythmic degradation rates, as long as these mediators and their substrate proteins follow certain conditions. We view this inherent biochemical mechanism as more basal than, yet conditionally synergistic to, the conventional circadian- or diurnal-controlled degradation mechanisms of the rhythmic degradation. Besides, our mathematical framework suggests that extra yet steady post-translational modifications (PTMs), such as with mono- or multisite phosphorylation, can elevate the rhythmicity of the substrate degradation rate and thereby cut down the proteosynthetic costs to substantial degrees. In addition, the model simulation accounts for the major trend of rhythmic ubiquitination and half-life data of animal and plant core clock proteins. The analysis of proteome-wide data is consistent with our expectations, as well. Our theory, aligned with the experimental data, provides a possibly prevailing mechanism of affording the price of daily biological rhythms in living creatures, and an evolutionary insight into the companionship between the circadian system and PTMs.

## Results

### Rhythmic degradation rates of rhythmically produced proteins can arise with steady post-translational regulation

If a protein not only is rhythmically produced but also decomposes with a rhythmic degradation rate, the complexity of the dynamics blurs our intuition and thus calls for a quantitative formulation and analysis. We derived a mathematical model that describes a temporal concentration profile of a protein with circadian production and degradation rates (see STAR Methods, computational modeling of protein ubiquitination without depending on other PTMs; for simplicity, the term “circadian” in this work will often refer to both circadian and diurnal). As a major route to protein degradation in eukaryotes, we here focus on ubiquitination, but our results are also applicable for other degradation mechanisms such as autophagy (STAR Methods, model application beyond protein ubiquitination). Although the incorporation of deubiquitinating processes is straightforward in our model, its outcome is rather condition-specific and hence will be omitted here.

**Figure 2:**
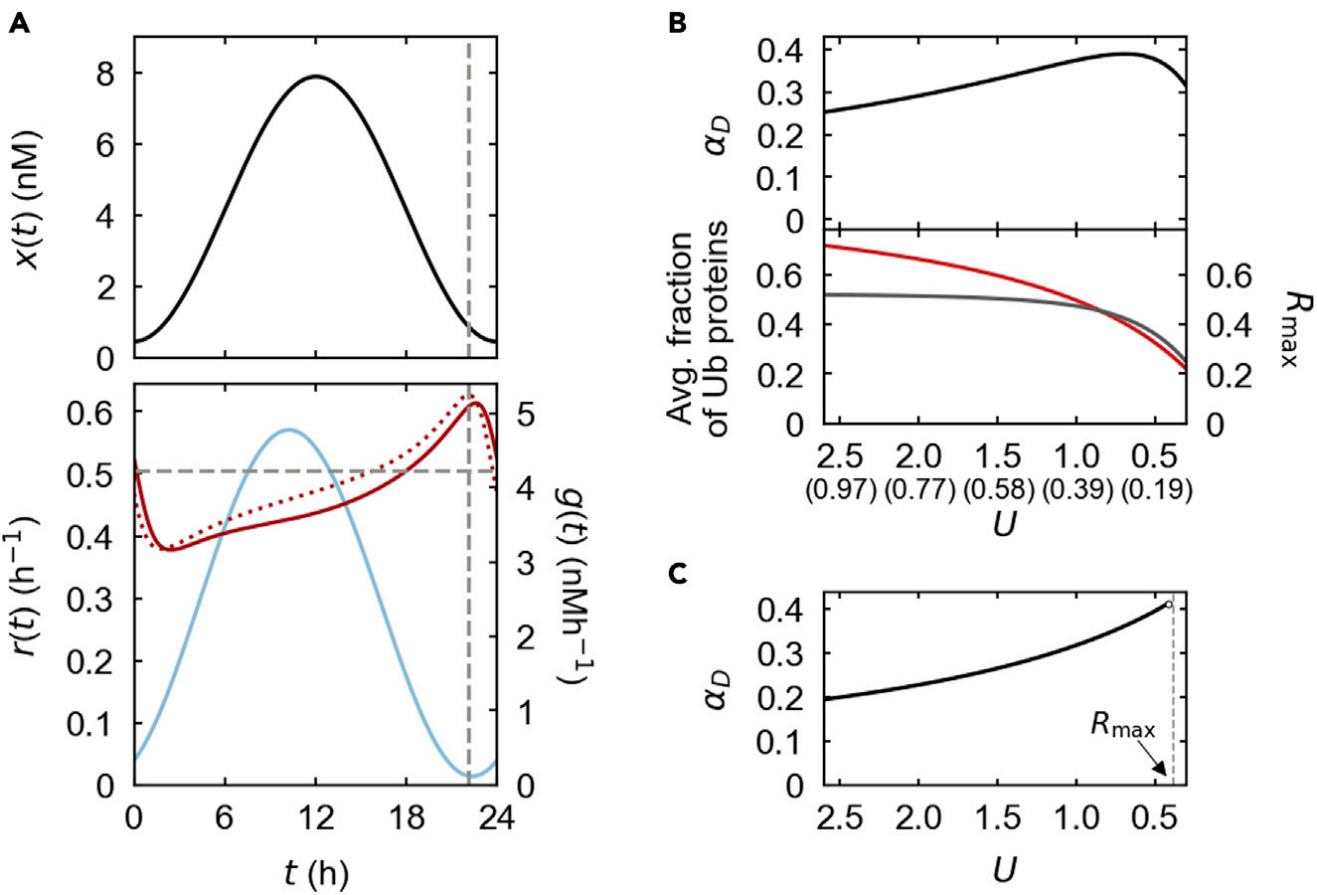
Phospho-independent degradation simulation with steady proteolytic mediators (A) The upper panel shows an example of simulated protein profile x(t) (black solid line). The lower panel shows the corresponding protein synthesis rate g(t) (light blue, solid line) and degradation rate r(t) (dark red, solid line). The estimate of r(t) based on Equation 1 is presented in the lower panel (dark red, dotted line; see also Figure S1). The minimum degradation rate (${\text{max}}_{t}\left[-x^{\prime }\left(t\right)/x\left(t\right)\right]$) under the assumption of a constant degradation rate is indicated in the lower panel (horizontal dashed line), together with the time point of the largest $-x^{\prime }\left(t\right)/x\left(t\right)$ in the upper and lower panels (vertical dashed lines) that determines the minimum constant degradation rate (STAR Methods, relationship between protein abundance profiles and biosynthetic costs). (B) Based on the same g(t) as in (A), we simulated the model by changing the concentration of ubiquitin ligases ($=\bar{u}$∝U), while fixing the other parameters. U denotes an appropriately scaled dimensionless measure of ubiquitinating activity, defined in STAR Methods, computational modeling of protein ubiquitination without depending on other PTMs. The simulation gave $\alpha_{D}$ in the upper panel and the time average of the fraction of ubiquitinated proteins (red solid line) and $R_{\text{max}}$ (${\text{max}}_{t}\left[-\left(1/r_{0}\right)\cdot x^{'}\left(t\right)/x\left(t\right)\right]$, gray solid line) in the lower panel. The corresponding $\bar{u}$ of each value of U is shown in the unit of nM in parenthesis under the horizontal axis. (C) This simulation strictly maintains the profile of x(t) in (A) and spans the whole range of U in (B), while fixing the other parameters and adjusting g(t) to each value of U. The resulting $\alpha_{D}$ is shown (black solid line) along with the boundary $U=R_{\text{max}}$ (gray dashed line). Biologically infeasible results are excluded here (see STAR Methods, computational modeling of protein ubiquitination without depending on other PTMs). For visual guidance, the horizontal axes in (B, C) are arranged in the descending order of U. The model in (A–C) consists of (Equation 6), (Equation 7), (Equation 8), (Equation 9), (Equation 10) in STAR Methods, computational modeling of protein ubiquitination without depending on other PTMs, and was simulated with the following parameter values: $\bar{u}=0.22$ nM (A), $\bar{v}=0$, $a_{0}=234.9$ nM^−1^h^−1^, $a_{1}=17,880.6$ h^−1^, $a_{2}=15,347.2$ h^−1^, $r_{0}=1.3$ h^−1^, and q=262.2 h^−1^.

Our model attributes the circadian production rate of a protein to a circadian mRNA expression or translation rate. Yet, a protein degradation rate in the model is not based on any explicitly time-dependent regulatory processes, but on constantly maintained proteolytic components such as the constant amount and activity of E3 ubiquitin ligases (STAR Methods, computational modeling of protein ubiquitination without depending on other PTMs). Nevertheless, clear circadian rhythmicity arises in the degradation rate coefficient over a range of biochemical conditions: Figure 2A exemplifies a 62.5% increase in the degradation rate from the lowest to the highest each day. This spontaneous rhythmicity can be understood by an unsynchronized interplay between protein translation and degradation processes. For example, in the case of protein ubiquitination, ubiquitin ligases with a finite binding affinity would not always capture all newly translated substrates, and therefore, a lower proportion of the substrates may be ubiquitinated during the rising phase of the substrate profile than during the falling phase. Hence, the degradation rate tends to be lower at times other than the falling phase. One may expect that this effect would be enhanced with a more limited level of ubiquitin ligases and their activity, under the condition when the substrate level with circadian production undergoes a steeply rising and falling oscillation. Here, we quantify the rhythmicity of the degradation rate by the peak-to-trough difference in the degradation rate divided by the peak degradation rate. This quantity, denoted by $\alpha_{D}$, has a larger value away from 0 and approaches 1 as the degradation rhythmicity becomes stronger (STAR Methods, quantification of degradation rhythmicity). As anticipated, the simulated decrease of ubiquitin ligase levels, while maintaining the same level of the circadian protein production, enhances the rhythmicity $\alpha_{D}$ by lowering the overall proportion of ubiquitinated proteins. However, this enhancement is very gradual over the range of the ubiquitin ligase levels (Figure 2B). Moreover, a further decrease of the ubiquitin ligases even slightly reduces $\alpha_{D}$ (Figure 2B). In fact, at a given protein production rate, a decrease in the ubiquitin ligases contributes both positively and negatively to the magnitude of $\alpha_{D}$: the negative effect is caused by the overall slowdown of protein turnover, which weakens the oscillation of the protein abundance (Figure 2B).

This intertwined relation between proteolytic activity levels and protein abundance oscillations motivated us to uncouple these two factors of degradation rhythmicity for its simpler and clearer analysis. Therefore, we devised a computational technique to simulate our model with a strictly maintained protein concentration profile, although parameter values are changed (STAR Methods, computational modeling of protein ubiquitination without depending on other PTMs). In this simulation, the protein production is not fixed but adapted to the parameter variations by adjusting the mRNA or translation-rate profile (STAR Methods, computational modeling of protein ubiquitination without depending on other PTMs). This technique rigorously controls for the effect of the protein abundance oscillation on the rhythmic degradation rate. Furthermore, the resulting simulation is expected to offer a more relevant evolutionary viewpoint, because the protein abundance profile directly influences a biological phenotype compared with an mRNA or translation-rate profile and thus represents a more fundamental position to which other elements in the system may have been adapted. For the remaining part of this work, we will apply this simulation technique.

Given a protein oscillation profile, the above new simulation does result in monotonically increasing rhythmicity $\alpha_{D}$, as the ubiquitin ligase amount or activity decreases (Figure 2C). For a better understanding of the model output, we derived an approximate formula for a protein degradation rate r(t) as a function of time t:

(Equation 1) $$r\left(t\right)\approx r_{0}{\left\{1+\frac{1}{U}\left[1+\frac{1}{r_{0}}\cdot \frac{x^{\prime }\left(t\right)}{x\left(t\right)}\right]\right\}}^{-1}$$

where x(t) denotes the protein concentration as a function of time, U denotes an appropriately scaled dimensionless measure of ubiquitinating activity that is proportional to the ubiquitin ligase concentration, and $r_{0}$ is the theoretical upper limit of the degradation rate for fully ubiquitinated proteins (STAR Methods, computational modeling of protein ubiquitination without depending on other PTMs). Equation 1 is fairly accurate in the anticipation of our simulation results (Figures 2A and S1A–S1I), although under rather specific biochemical conditions (STAR Methods, computational modeling of protein ubiquitination without depending on other PTMs). Even beyond such biochemical conditions, Equation 1 can still be useful to provide analytical insights into the system.

It is clear from Equation 1 that a rhythmic degradation rate naturally emerges from the oscillation of protein concentration x(t). We further define $R_{\text{max}}$ and $R_{\text{min}}$ as the maximum and minimum values of $R\left(t\right)\equiv -(1/r_{0})\cdot x^{'}\left(t\right)/x\left(t\right)$ of the day, respectively, and their magnitudes are proportional to the highest decline and rising rates of the logarithmic protein abundance. In agreement with Equation 1, the simulated degradation rate consistently peaks when $R\left(t\right)\approx R_{\text{max}}$, that is, near the trough of the protein level during its falling phase (Figure 2A). At this time point, the largest fraction of the proteins has become tagged with ubiquitin without much addition of newly translated proteins.

Based on Equation 1, we mathematically showed that a ubiquitin ligase concentration cannot be lower than a certain limit (specifically, $U≳R_{\text{max}}$) in order to maintain a given oscillatory profile of the substrate (STAR Methods, computational modeling of protein ubiquitination without depending on other PTMs); otherwise, the substrate protein is forced to have a weaker oscillation with a smaller $R_{\text{max}}$. This mathematical finding is highly consistent with the results of our model simulation with a given protein profile, where U could not be reduced below $R_{\text{max}}$ without the loss of biological feasibility (e.g., Figure 2C). As a result, the rhythmicity $\alpha_{D}$ is maximized at a point that U is reduced at most to near $R_{\text{max}}$ (e.g., Figure 2C).

The dependence of rhythmicity $\alpha_{D}$ on proteolytic factors and the substrate oscillation can be systematically assessed by the following formula based on Equation 1 (STAR Methods, computational modeling of protein ubiquitination without depending on other PTMs):

(Equation 2) $$\alpha_{D}\approx \frac{R_{\text{max}}-R_{\text{min}}}{U+1-R_{\text{min}}}$$

This $\alpha_{D}$ is further approximated as $\alpha_{D}\approx 2R_{\text{max}}/\left(U+1+R_{\text{max}}\right)\;$ if the protein profile is approximately symmetric around its peak phase and so $R_{\text{min}}\approx -R_{\text{max}}$ in Equation 2. This form of $\alpha_{D}$ reveals the quantitative structure of the degradation's rhythmicity, which is enhanced by a reduction in the ubiquitin ligases (U) and an increase in the substrate oscillation ($R_{\text{max}}$). Our extensive model simulation of various protein profiles, ubiquitin ligase concentrations, and kinetic parameter values shows that the quantitative relation in Equation 2 works correctly under the conditions where Equation 1 is likely valid (Figure S1). Yet, even stronger degradation rhythmicity ($\alpha_{D}≳0.7$) than expected by Equation 2 is seen in physiologically relevant conditions (Table S1). For a part of the conditions not covered by Equations 1 and 2, we derived the alternative form of r(t):

(Equation 3) $$r\left(t\right)\approx r_{0}{\left\{1+\frac{1}{U}\left[1-R\left(t\right)\right]\left[1+\frac{r_{0}}{q}U+\frac{a_{0}}{a_{1}+q}x\left(t\right)\right]\right\}}^{-1}$$

where $a_{0}$ ($a_{1}$) denotes the binding (unbinding) rate of proteins and ubiquitin ligases, q denotes the ubiquitination rate of proteins that are binding to ubiquitin ligases, and x(t), $R\left(t\right),r_{0}$, and U are the same as the previous ones (STAR Methods, computational modeling of protein ubiquitination without depending on other PTMs). The fraction $a_{0}x\left(t\right)/\left(a_{1}+q\right)$ in this formula originates from Michaelis-Menten kinetics of the protein binding to ubiquitin ligases, but its effect is deflected by the temporal profile of 1−R(t). If |R(t)|≪1 and $U\ll \frac{a_{0}q}{\left(a_{1}+q\right)\left(r_{0}+q\right)}x\left(t\right)$, Equation 3 resembles the degradation-rate formula assumed in Goldbeter (1996) and Kurosawa and Iwasa (2002). Equation 3 shows clear agreement with the simulation results in the relevant biochemical conditions, as exemplified by Figures S1J–S1L.

### Extra PTMs can facilitate the rhythmicity of protein degradation and biosynthetic cost reduction

Thus far, we have considered a rather simple scenario where ubiquitin ligase activation does not require any prior substrate modifications. In nature, however, cross talk between ubiquitination and other PTMs is prevalent (Filipčík et al., 2017; Hunter, 2007). Therefore, we now consider the case where protein phosphorylation promotes subsequent ubiquitination. This modeling approach and its conclusions can also be applied to modifications other than phosphorylation (STAR Methods, model application for PTMs beyond phosphorylation).

For the modeling of phosphorylation-dependent ubiquitination, we considered constant levels and activities of protein kinases, still without any explicitly time-dependent, post-translational processes. In this modified model, mono- or multisite phosphorylation is a prerequisite for ubiquitination (STAR Methods, computational modeling of phosphorylation-dependent protein ubiquitination). Although more than one degradation route may exist for the same substrate with multiple phosphorylation events, most of these simulation results are largely reflective of the effects of individual phospho-specific degradation pathways (Figure S2 and STAR Methods, model expansion for multiple degradation routes); therefore, we here focus on the streamlined individual pathways.

**Figure 3:**
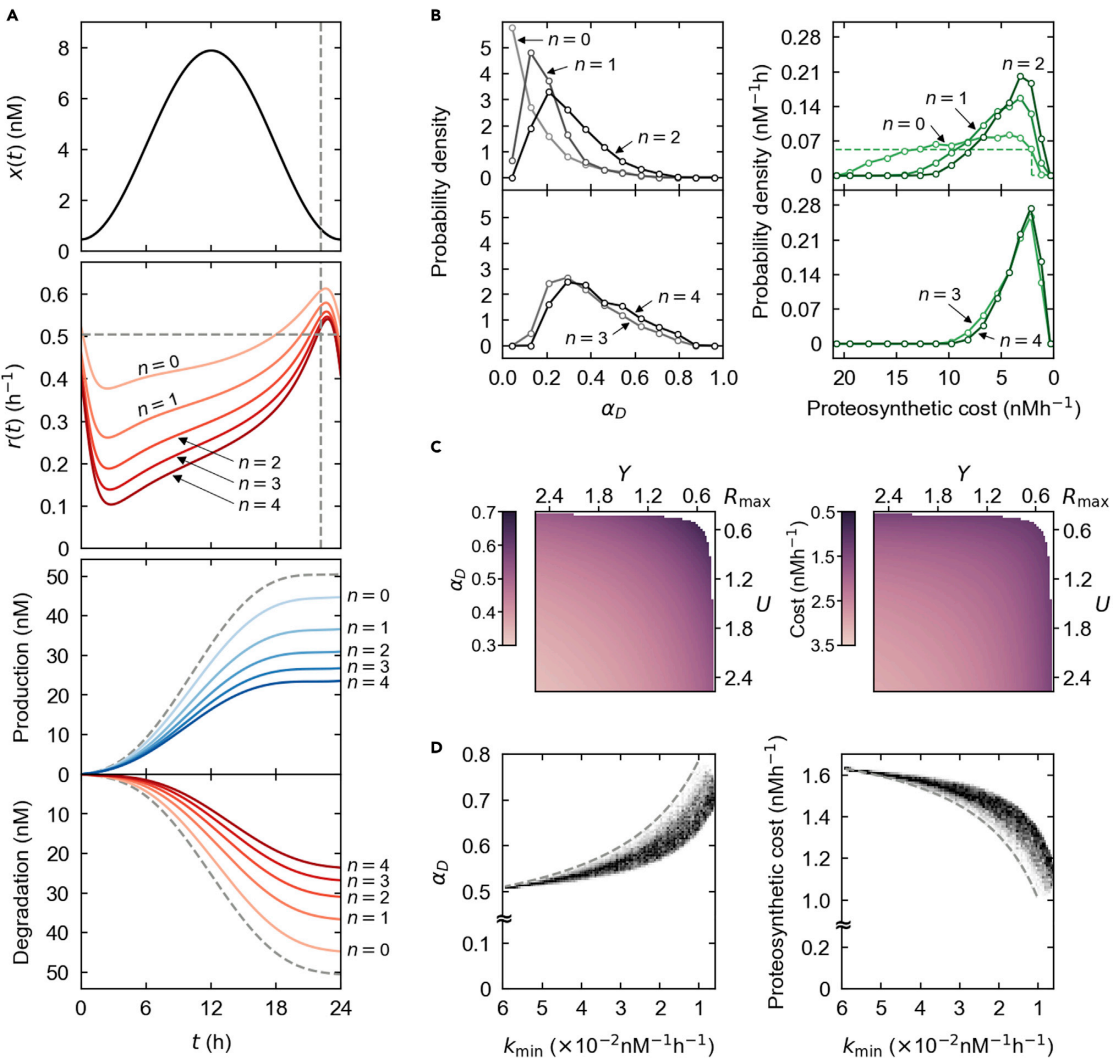
Phospho-dependent degradation simulation with steady proteolytic mediators n denotes the number of phosphorylation events required for protein ubiquitination. n=0 corresponds to phospho-independent ubiquitination. (A) Maintaining the protein profile x(t) and model parameters in Figure 2A, we increased n and simulated the degradation rate r(t) for each n (solid lines in the center panel). The bottom panel shows the total protein amount produced (bluish solid line) or degraded (reddish solid line) over time for each n. In the top and center panels, horizontal and vertical dashed lines are the same in Figure 2A with a constant degradation rate. The dashed lines in the bottom panel correspond to this constant degradation-rate case. (B) Given the protein profile x(t) in (A), we computed the probability distributions of $\alpha_{D}$ and a proteosynthetic cost for each n (solid lines) over physiologically relevant parameter ranges in Table S1. The dashed line in the right upper panel corresponds to a constant degradation-rate case. (C) When n=1, $\alpha_{D}$ and proteosynthetic costs are shown with varying kinase (∝Y) and ubiquitin ligase (∝U) concentrations, while the other parameters and x(t) are fixed as (A). The top-right corner of each plot corresponds to $Y=U=R_{\text{max}}$. Biologically infeasible regimes are not plotted here (STAR Methods, computational modeling of phosphorylation-dependent protein ubiquitination; see also Figure S3). (D) $k_{\text{min}}$ denotes the lowest binding rate of a kinase across phosphosites of a target protein. When n=3, the density plot of varying $k_{\text{min}}$ and the corresponding $\alpha_{D}$ was obtained over randomly sampled kinase binding rates from physiologically relevant ranges in Table S1 (the other parameters and x(t) were fixed as in (A)). Likewise, the density plot of varying $k_{\text{min}}$ and the corresponding costs was obtained. The dashed lines indicate the results of the identical binding rates across the phosphosites. Figure S4 provides additional information. For visual guidance, the costs, Y, and U in (B, C) and $k_{\text{min}}$ in (D) are arranged in the descending order. For notations here, refer to STAR Methods, computational modeling of protein ubiquitination without depending on other PTMs and computational modeling of phosphorylation-dependent protein ubiquitination. The models in (A–D) consist of (Equation 6), (Equation 7), (Equation 8), (Equation 9), (Equation 10) and (Equation 19), (Equation 20), (Equation 21), (Equation 22), (Equation 23), (Equation 24), (Equation 25), (Equation 26) in STAR Methods, computational modeling of protein ubiquitination without depending on other PTMs and computational modeling of phosphorylation-dependent protein ubiquitination with the following parameter conditions: y=152.7 nM (A, D), z=0, $k=k_{1}=k_{2}=k_{3}=k_{4}=0.013$ nM^−1^h^−1^ (A, C), and $k_{1}=k_{2}=k_{3}=k_{4}$ (B). The other parameters are the same as in Figure 2.

For a given protein abundance profile, phospho-dependent ubiquitination in most of our model results tends to confer higher rhythmicity on the degradation rate than the previously simulated, phospho-independent ubiquitination (Figure 3A). In most simulation cases, the more the number of phosphoryl group attachments required for ubiquitination, the stronger the rhythmicity that tends to develop until eventually saturated (e.g., Figures 3A and 3B; in the case of Figure 3B, the distribution of $\alpha_{D}$ roughly converges around tri- or tetra-phosphorylation). For example, a peak-to-trough ratio of the degradation rate in Figure 3A increases by 36.3%–220.7% with mono- to tetra-phosphorylation, compared with phospho-independent degradation. In the case of Figure 3A, the degradation's rhythmicity enhanced by phosphorylation can be explained as follows: kinases with a finite binding affinity cannot always achieve full phosphorylation of all newly translated proteins, and this effect retards further ubiquitination depending on the number of the phosphorylation events required for ubiquitination. This retardation is prominent during the rising phase of the protein abundance, where new proteins are actively accumulated. This phase largely overlaps with the aforementioned phase of a low protein degradation rate under phospho-independent ubiquitination. On the other hand, this retardation effect by phosphorylation diminishes near the trough of the protein profile during the falling phase, where new protein translation is slow and the declining amount of proteins is subject to saturating phosphorylation and ubiquitination without much lag. Intriguingly, this moment coincides with the previously mentioned peak time of a protein degradation rate under phospho-independent ubiquitination. To summarize, the retardation of ubiquitination due to phosphorylation leads to a further drop in a low degradation rate, exacerbated by the presence of more phosphorylation events, but does not much impact a peak degradation rate. This time-of-day-differential effect enhances the rhythmicity of the degradation rate, which is manifested by more phosphorylation events prior to ubiquitination. The detailed behavior rather varies and can be conditionally different from Figure 3A, but the overall enhancement of the degradation rate's rhythmicity by phosphorylation still remains valid for most simulation cases (e.g., Figure 3B).

These extra PTMs in a proteolytic pathway serve as a simple mechanism for proteosynthetic cost reduction, via the enhanced rhythmicity in the protein degradation rate as well as via the reduction of the overall degradation rate. The rhythmic degradation rate becomes selectively high at the falling phase of the protein profile but stays markedly lower elsewhere, saving much of the gross protein loss and thereby the protein synthetic cost (STAR Methods, relationship between protein abundance profiles and biosynthetic costs). Given a particular protein abundance profile, Figure 3A demonstrates an 18.1%–47.3% reduction in protein synthesis with mono- to tetra-phosphorylation, compared with phospho-independent degradation. Noteworthy is that rhythmic degradation by ubiquitination alone, not dependent on phosphorylation, saves 11.4% in a proteosynthetic cost in the case of Figure 3A, compared with a constant degradation rate; therefore, phospho-dependent ubiquitination saves additional costs from this base cost. In most of our simulated conditions, the greater the number of phosphorylation events, the less the proteosynthetic costs tend to be incurred, until they converge to certain ranges (e.g., Figure 3B; in the case of Figure 3B, the cost distribution roughly converges around tri- or tetra-phosphorylation). One may wonder whether the expense of adenosine triphosphate (ATP) hydrolysis for phosphorylation discounts the energetic benefit of the proteosynthetic cost reduction. However, this discounting effect would be negligible in practice even in the case of multisite phosphorylation. This is due to the fact that the formation of each peptide bond in protein synthesis requires the release of at least four phosphoryl groups from ATP and guanosine triphosphate (GTP) molecules (Urry et al., 2016), so the net free energy is always saved as long as ≳n/4N of the protein synthesis is reduced with the phospho-dependent ubiquitination (N is the number of amino acids in a substrate protein and n is the number of the phosphorylation events for the ubiquitination). The calculation with N≳100 and n≲4 suggests that a mere ∼1% reduction in the protein synthesis under the phospho-dependent ubiquitination would be sufficient to pay off the ATP expense for the phosphorylation, which then does not serve as a meaningful factor in the net energetic cost.

Many circadian clock proteins have been reported as the targets of phosphorylation (Farré and Kay, 2007; Reischl and Kramer, 2011; Wang et al., 2010; Zhou et al., 2015). By our count, two-thirds of the core clock proteins in plant *Arabidopsis thaliana* are known phosphoproteins (STAR Methods, known phosphoproteins in the Arabidopsis circadian clock). Apart from other functions of protein phosphorylation such as protein localization and protein complex formation (Reischl and Kramer, 2011; Wang et al., 2010), the present outcomes from our model suggest that phosphorylation helps lower biosynthetic costs by facilitating the rhythmic turnover of circadian proteins and hence relieve the price of daily biological rhythms. Later, we will revisit this issue with additional analysis.

Analogous to the previous case of phospho-independent ubiquitination, a decrease in the amount of protein kinases or ubiquitin ligases under phospho-dependent ubiquitination monotonically increases the degradation rhythmicity $\alpha_{D}$ (e.g., Figures 3C and S3). The increased rhythmicity $\alpha_{D}$ is accompanied by a reduced proteosynthetic cost, as well (e.g., Figures 3C and S3). In the case of mono-phosphorylation, the following approximate formula exists for the degradation rate r(t) in a similar fashion to Equation 1:

(Equation 4) $$r\left(t\right)\approx r_{0}{\left\{\left\{1+\frac{1}{U}\left[1-R\left(t\right)\right]\right\}\left[1-\frac{1}{Y}R\left(t\right)\right]+\frac{1}{Y}\right\}}^{-1}$$

where Y denotes an appropriately scaled dimensionless measure of kinase activity that is proportional to the kinase concentration, $R\left(t\right)\equiv -(1/r_{0})\cdot x^{'}\left(t\right)/x\left(t\right)$ as defined above, and x(t), $r_{0}$, and U are the same as in Equation 1 (STAR Methods, computational modeling of phosphorylation-dependent protein ubiquitination). Equation 4 becomes equivalent to Equation 1 when the kinases are very abundant and thus not an appreciable limiting factor in the degradation pathway (usually when Y≫U). Equation 4 reveals that a finite level of the kinases amplifies the degradation rhythmicity (which originates from the oscillation of R(t)), compared with the degradation in Equation 1 that does not depend on prior phosphorylation.

With previously defined $R_{\text{max}}$, we mathematically showed that the concentration of kinases, as well as that of ubiquitin ligases, cannot be lower than a certain limit, i.e., $Y,\;U≳R_{\text{max}}$ in order to maintain a given protein profile (STAR Methods, computational modeling of phosphorylation-dependent protein ubiquitination). Otherwise, the protein would show a weaker oscillation with a smaller $R_{\text{max}}$ in force, in a similar way to the previous analysis of Equation 1. Consistently, our model simulation shows that both Y and U could not be reduced below $R_{\text{max}}$, and the degradation rate ends up with its largest rhythmicity (and the proteosynthetic cost is minimized) when Y and U are reduced at most to near $R_{\text{max}}$ (Figure 3C).

The expansion of Equation 4 to cover the ubiquitination with multisite phosphorylation is presented in STAR Methods, computational modeling of phosphorylation-dependent protein ubiquitination. Briefly, the expanded formula suggests that, among the kinase binding rates across multiple phosphosites, the lowest binding rate determines the overall possible ranges of the rhythmicity $\alpha_{D}$ and of the proteosynthetic cost. This expectation is supported by our simulation results (e.g., Figures 3D and S4).

In our model, we further considered the activities of phosphatases and deubiquitinating enzymes, but their effects on the degradation rhythmicity were condition-dependent (STAR Methods, computational modeling of phosphorylation-dependent protein ubiquitination).

To summarize, our results suggest that a rhythmic degradation rate of a circadian protein does not necessarily require rhythmic post-translational regulation, while extra, constant PTMs (including phosphorylation and other modifications coverable by our model as in STAR Methods, model application for PTMs beyond phosphorylation) can further enhance the rhythmicity of the protein degradation and reduce proteosynthetic costs. Here, the degradation rate is escalated during the falling phase of the protein oscillation. Therefore, we expect that this inherent biochemical mechanism may also synergize with conventional circadian- or diurnal-regulated degradation mechanisms (Cha et al., 2017; Kiba et al., 2007; Lamia et al., 2009; Toledo et al., 2018) to amplify the degradation rhythmicity, if that circadian-regulated degradation preferably targets the protein falling phase.

### TIMELESS shows a ubiquitination pattern consistent with the model prediction

We observe that the rhythmic fraction of ubiquitinated proteins in our simulation tends to have an almost single peak at the time of the near-maximum $-x^{\prime }\left(t\right)/x\left(t\right)$, when the protein level x(t) is approximately sinusoidal. In the example of the protein profile x(t) in Figure 3A and the parameter conditions in Figure 3B, all the simulation results with $\alpha_{D}>0.2$ through phospho-independent and mono- to tetra-phosphorylation-dependent cases showed almost single peaks of ubiquitinated protein fractions, only with < 2-h peak-time differences from the $-x^{\prime }\left(t\right)/x\left(t\right)$ profile. This tendency is analytically supported by Equations 17, 18, and 29 (equivalent to Equations 1, 3, and 4), as well.

**Figure 4:**
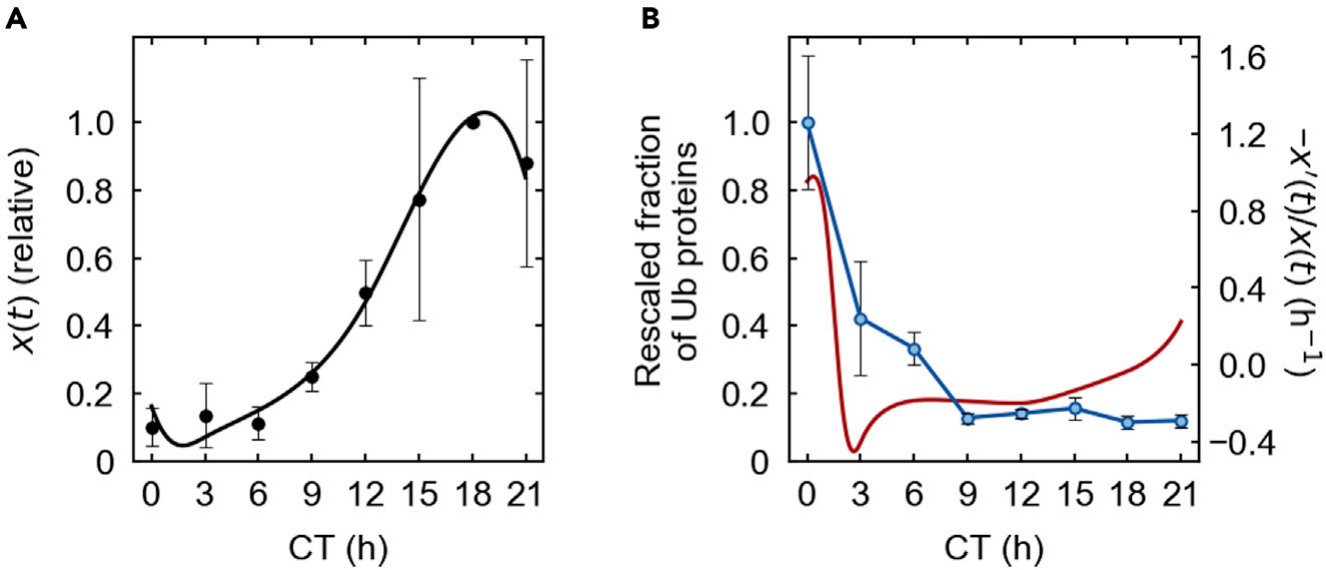
TIM abundance and ubiquitination data (A) The experimental abundance of TIM at each time t (CT). The profile x(t) (solid line) is the spline of the abundance data points (STAR Methods, analysis of TIM ubiquitination data). (B) The experimental fraction of ubiquitinated TIM (blue), together with $-x^{\prime }\left(t\right)/x\left(t\right)$ (red) at each time t (CT). The average of the ubiquitinated fractions at CT 0 h is scaled to 1 (STAR Methods, analysis of TIM ubiquitination data). In (A, B), each data point and error bar represent the average and standard deviation of two replicates, respectively.

TIMELESS (TIM) is one of the core proteins in the circadian clock of *Drosophila melanogaster* (Sehgal et al., 1994; Szabó et al., 2018). Both its oscillatory protein level and ubiquitinated protein ratio in a relative manner are available over time from the recent experiments with *Drosophila* in constant darkness (Szabó et al., 2018), as presented in Figures 4A and 4B (STAR Methods, analysis of TIM ubiquitination data). Figure 4B demonstrates that the fraction of ubiquitinated TIM markedly peaks at the phase between circadian time (CT) 21 h (= −3 h) and 3 h, which is near the estimated peak phase of $-x^{\prime }\left(t\right)/x\left(t\right)$ (CT 0.35 h). In view of the aforementioned simulation outcomes, our theory then at least captures the observed feature of TIM ubiquitination, although this consistency may not necessarily rule out alternative explanations of these TIM data.

### Plant and mammalian clock proteins exhibit rhythmic degradation patterns with low biosynthetic costs

**Figure 5:**
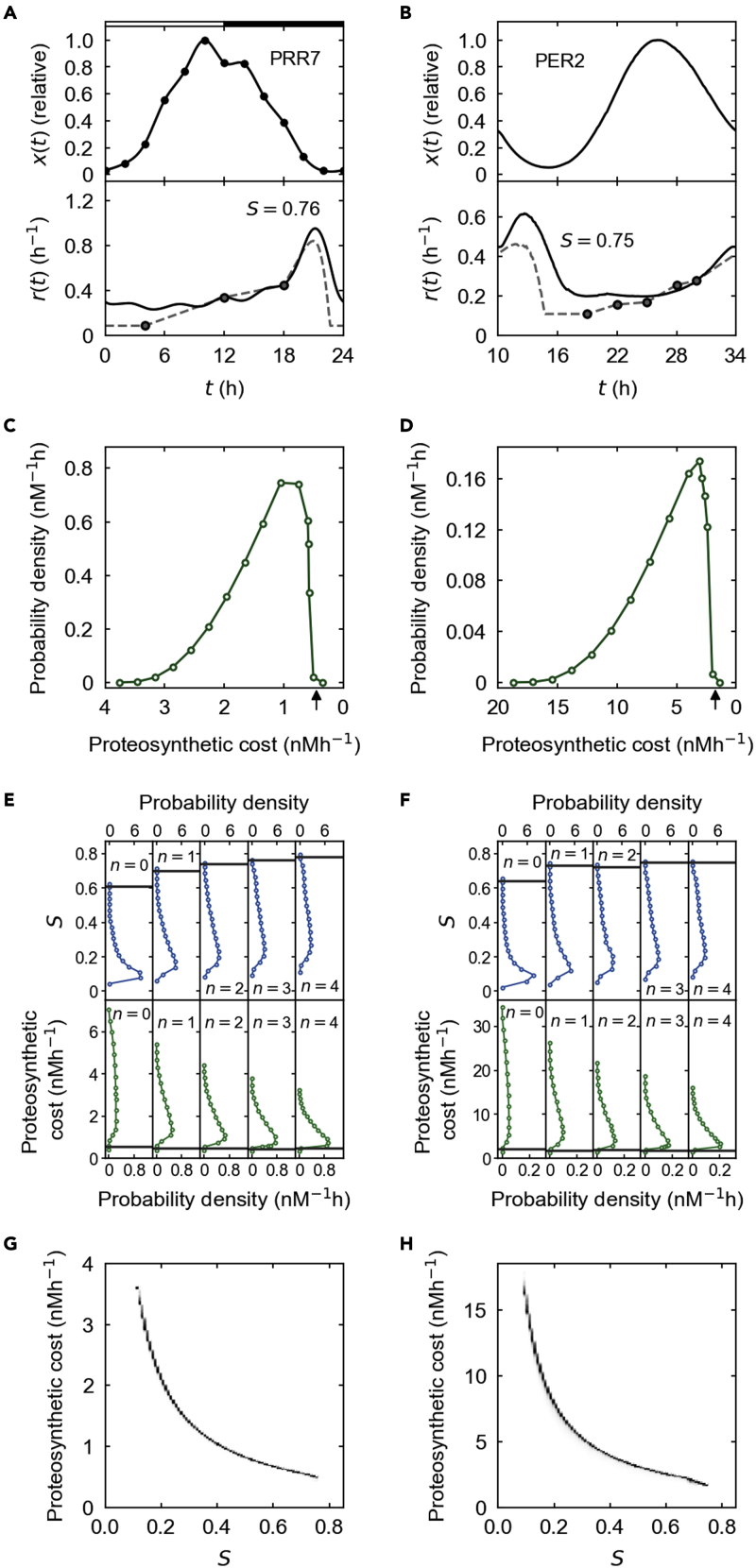
PRR7 and PER2 degradation simulation n denotes the number of phosphorylation events required for protein ubiquitination. n=0 corresponds to phospho-independent ubiquitination. (A and B) The upper panel shows the experimental abundance profile of PRR7 (A) or PER2 (B). The lower panel shows the simulated (solid line, when n=3) and empirical (dashed line) degradation-rate profiles, together with the observed degradation-rate data (circles). S in the lower panel is the similarity between the simulated and empirical degradation-rate profiles (see STAR Methods, PRR7 and PER2 degradation modeling and analysis). (C and D) The probability distribution of a proteosynthetic cost of PRR7 (C) or PER2 (D) from uniformly sampled parameter sets, when n=3. An arrow indicates the proteosynthetic cost of a sampled parameter set with the largest S. For visual guidance, the horizontal axis is arranged in the descending order of a proteosynthetic cost. (E and F) The probability distributions (green solid line) of S and a proteosynthetic cost of PRR7 (E) or PER2 (F) for each n from uniformly sampled parameter sets. Horizontal dim gray lines indicate the largest S values (upper panel) and their associated proteosynthetic costs (lower panel). See also Table S2. (G and H) The density plot of S and a proteosynthetic cost of PRR7 (G) or PER2 (H) from uniformly sampled parameter sets, when n=3. The densities were normalized to the highest density for each range of S. The model results in (A–H) are based on (Equation 6), (Equation 7), (Equation 8), (Equation 9), (Equation 10) and (Equation 19), (Equation 20), (Equation 21), (Equation 22), (Equation 23), (Equation 24), (Equation 25), (Equation 26) in STAR Methods, computational modeling of protein ubiquitination without depending on other PTMs and computational modeling of phosphorylation-dependent protein ubiquitination with parameter conditions z=0, $\bar{v}=0$, and $k_{1}=k_{2}=k_{3}=k_{4}$. A total of 10^6^ sets of parameter values (C–H) were uniformly sampled from physiologically relevant ranges in Table S1 (see STAR Methods, PRR7 and PER2 degradation modeling and analysis and Figure S5 for further details).

We now move to the examination of the observed half-lives of plant and mammalian clock proteins. PSEUDO RESPONSE REGULATOR 7 (PRR7) and PERIOD2 (PER2) proteins are core constituents in the *Arabidopsis* and mouse clocks, respectively, and known to exhibit both rhythmic concentrations and half-lives over time (Farré and Kay, 2007; Jo et al., 2018; Nakamichi et al., 2010; Zhou et al., 2015). Figures 5A and 5B show these experimental concentration profiles and degradation rates (degradation rate and half-life are inversely proportional to each other). To what extent is our proposed mechanism of rhythmic degradation able to capture the observed patterns of these degradation rates?

Reportedly, both PRR7 and PER2 are phosphoproteins and undergo proteasome-mediated degradation, and the PER2 degradation involves the phospho-specific recognition by a ubiquitin ligase complex subunit β-transducin repeat-containing protein (β-TrCP) (Farré and Kay, 2007; Zhou et al., 2015). For PRR7, it is unclear at the moment whether phosphorylation affects protein stability. For PRR7 and PER2, we considered our present model of ubiquitination with or without phosphorylation dependency. Yet, as previously noted, our model is applicable for a number of PTMs that can precede ubiquitination, rather than only for phosphorylation (STAR Methods, model application for PTMs beyond phosphorylation). This point would be particularly relevant to the less-well-characterized PRR7 degradation. The PRR7 and PER2 abundance profiles in the simulation were maintained as strictly the same as their own experimental profiles, based on the computational technique used in our above simulations (STAR Methods, computational modeling of protein ubiquitination without depending on other PTMs and computational modeling of phosphorylation-dependent protein ubiquitination; Figures 5A and 5B). Although actual PER2 degradation involves the combination of multiple pathways (Liu et al., 2018; Zhou et al., 2015), our model with simplified pathways aimed to capture the effective mode of the PER2 degradation without losing the interpretability of the results by unnecessary model complexity (STAR Methods, PRR7 and PER2 degradation modeling and analysis). Nevertheless, a full expansion of the model for realistic PER2 degradation, as well as the consideration of phosphatases and deubiquitinating enzymes in the PER2 and PRR7 simulation, does not much affect the results presented here (Figure S5 and STAR Methods, PRR7 and PER2 degradation modeling and analysis). We made our PRR7 and PER2 simulation more compact by harnessing the previous finding in Figures 3D and S4 that the lowest kinase binding rates in multisite phosphorylation guide the overall substrate degradation dynamics (STAR Methods, PRR7 and PER2 degradation modeling and analysis).

Within a physiologically relevant range, we uniformly searched through the sets of biochemical parameter values for the simulations closely mimicking the experimental degradation patterns (Table S1 and STAR Methods, PRR7 and PER2 degradation modeling and analysis). More specifically, given the scarcity of experimental PRR7 and PER2 degradation rates, we temporally extended (interpolated and extrapolated) these data points in a reasonable way (STAR Methods, PRR7 and PER2 degradation modeling and analysis) and measured similarity S between this empirical degradation-rate profile and the simulated degradation-rate profile from each parameter set. Here, S was devised to approach 1 away from 0, as the simulated profile quantitatively better matches the empirical profile (STAR Methods, PRR7 and PER2 degradation modeling and analysis). Figures 5A and 5B show simulated PRR7 and PER2 degradation rates with S= 0.76 and 0.75 compared with their empirical profiles, respectively.

According to our previous analysis, proteosynthetic cost reduction may benefit considerably from rhythmic protein degradation. To check this effect, we computed proteosynthetic costs in addition to the degradation rates (STAR Methods, relationship between protein abundance profiles and biosynthetic costs). Surprisingly, we found that, in mono- to tetra-phosphorylation-dependent and phospho-independent degradation simulations, the degradation-rate profiles closest to the empirical ones (i.e., with the largest S values) incur only the proteosynthetic costs comparable with the minimum costs achievable by the simulations (Figures 5C–5F): the minimum costs were just ≲10% and ≲15% different from the costs of the largest S values, with uniformly sampled parameter sets and parameter optimization, respectively ($P<1.2\times 10^{-4}$; STAR Methods, PRR7 and PER2 degradation modeling and analysis and quantification and statistical analysis and Table S2). For example, in the case of tri-phosphorylation, there exist PRR7 and PER2 parameter sets with the costs from which the minimum costs under uniform parameter sampling were only 3.5% and 2.2% different, respectively. These PRR7 and PER2 parameter sets gave the most realistic degradation-rate profiles with S= 0.76 and 0.75 in the case of tri-phosphorylation, respectively (Figures 5C and 5D).

Consistently, we found the very strong tendency that the more similar a computed degradation-rate profile is to the empirical profile, the less the proteosynthesis tends to cost in the mono-to tetra-phosphorylation and phospho-independent cases (e.g., Spearman's ρ<−0.99 between S and the proteosynthetic cost in Figures 5G and 5H for the tri-phosphorylation case and $P<10^{-4}$; see STAR Methods, quantification and statistical analysis). In other words, lowering the biosynthetic costs under physiological conditions can account for the substantial quantitative trends of the observed PRR7 and PER2 degradation rates. Together, these results are consistent with our expectation on the biosynthetic cost savings promoted by the rhythmic degradation of circadian clock proteins.

Compared with phospho-independent degradation, phospho-dependent degradation tends to lower the overall proteosynthetic costs from sampled parameters in the PRR7 and PER2 simulation (Figures 5E and 5F), reminiscent of our previous simulation results in Figure 3B. The phospho-dependent degradation tends to give better S values as well (Figures 5E and 5F and Table S2). The overall costs and S values, however, show only slight or essentially no improvements once the number of phosphorylation events passes about two (Figures 5E and 5F). Compared with the phospho-independent scenario, the enhanced S values in the phospho-promoted PRR7 degradation indicate that PRR7 degradation in nature may be indeed mediated by prior PTMs such as phosphorylation (Farré and Kay, 2007), warranting further experiments. Some discrepancies between the simulated and experimental degradation rates in Figures 5A and 5B may be due to possibly missing components in our model, such as nucleocytoplasmic partitioning of molecular events or partly circadian-regulated degradation processes.

### Circadian proteome data correlate phosphorylation with the need for biosynthetic cost saving

**Figure 6:**
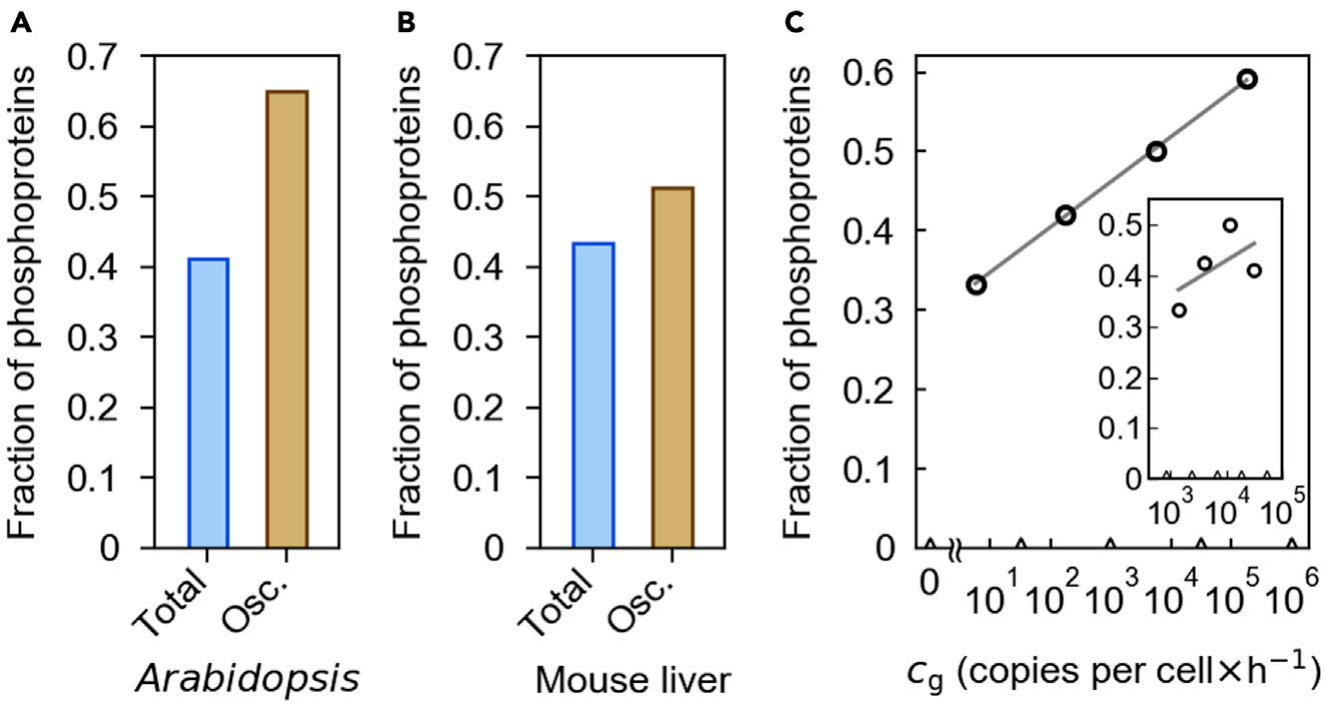
Analysis of *Arabidopsis* and mouse-liver proteome data (A and B) The fraction of phosphoproteins in either all detected proteins (“Total”) or the proteins with circadian oscillations (“Osc.”) is shown for *Arabidopsis* (A) or mouse liver (B). $P<10^{-4}$ in (A) and P=0.01 in (B) (STAR Methods, analysis of Arabidopsis and mouse-liver proteome data and quantification and statistical analysis). (C) The fraction of phosphoproteins (circle) in mouse-liver circadian proteins is plotted across different ranges of $c_{\text{g}}$ (STAR Methods, analysis of Arabidopsis and mouse-liver proteome data). Each range of $c_{\text{g}}$ for the calculation of the phosphoprotein fraction lies between adjacent triangular ticks on the horizontal axis. A linear regression line is drawn for visual guidance (gray solid line; $R^{2}=0.9992$). P=0.008 (STAR Methods, quantification and statistical analysis). The inset shows the phosphoprotein fractions (circles) in a similar way, but only includes the set of phospho- and non-phospho-proteins in the controlled abundance range described in STAR Methods, analysis of Arabidopsis and mouse-liver proteome data. In the inset, each range of $c_{\text{g}}$ for the calculation of the phosphoprotein fraction lies between adjacent triangular ticks on the horizontal axis, and a linear regression line is drawn for visual guidance (gray solid line; $R^{2}=0.33$). P=0.04 (STAR Methods, quantification and statistical analysis). (C) and its inset have the same aspect ratio of scales on the horizontal and vertical axes.

Our model proposes that additional PTMs can noticeably lower the biosynthetic costs of proteins with circadian abundances through enhanced degradation rhythmicity (Figures 3A and 3B). Not only would this proposed effect be relevant to the core clock proteins examined thus far, but possibly also to a broad repertoire of circadian-regulated proteins. We therefore expected that, at the proteomic level, circadian-regulated proteins are more likely targets of the PTMs than non-circadian proteins, considering the biological benefits of reducing the substantial cost of the circadian oscillations. Because protein phosphorylation has been reported as a major type of the PTM that triggers ubiquitination for the protein degradation (Filipčík et al., 2017; Hunter, 2007), we evaluated the enrichment of phosphoproteins in circadian proteomes in *Arabidopsis* and mouse liver. Our analysis of *Arabidopsis* protein data from Choudhary et al. (2016) and other datasets in combination reveals that 65.1% of the proteins with circadian abundances are phosphoproteins, surpassing the fraction of phosphoproteins (41.2%) among all detected *Arabidopsis* proteins (Figure 6A and $P<10^{-4}$; see STAR Methods, analysis of Arabidopsis and mouse-liver proteome data and quantification and statistical analysis).

Similarly, mouse liver data combined from Mauvoisin et al. (2014) and other sources show the enrichment of phosphoproteins in the proteins with circadian abundances, but to a lesser degree than the *Arabidopsis* data: 51.3% and 43.4% for the phosphoprotein fractions among the circadian and entire mouse liver proteins, respectively (Figure 6B and P=0.01; STAR Methods, analysis of Arabidopsis and mouse-liver proteome data and quantification and statistical analysis). The only moderate difference between these two fractions in the mouse liver data prompted us to ask whether the mouse liver phosphoproteins with circadian oscillations are biased toward proteins of otherwise higher biosynthetic costs. In the previous study, we introduced quantity $c_{\text{g}}$, the lower limit of the proteosynthetic cost of a given protein when its degradation rate is assumed to be constant (Jo et al., 2018). The quantity $c_{\text{g}}$ of each circadian protein is estimated to be the maximum decline rate of the logarithmic protein abundance (i.e., the maximum of $-x^{\prime }\left(t\right)/x\left(t\right)$) multiplied by the protein's typical absolute abundance level (STAR Methods, analysis of Arabidopsis and mouse-liver proteome data). The quantity $c_{\text{g}}$ can be roughly viewed as the characteristic scale of the cost when the degradation rate is not rhythmic enough. In this regard, $c_{\text{g}}$ indicates potential biological need for a highly rhythmic degradation rate to reduce this cost level. We hence expected that high $c_{\text{g}}$ proteins would be the preferable targets of phosphorylation among the circadian proteins in the mouse liver.

Indeed, our analysis of the mouse liver data reveals that the higher the $c_{\text{g}}$ values, the more is the proportion of phosphoproteins with circadian oscillations, as shown in Figure 6C (P=0.008; STAR Methods, analysis of Arabidopsis and mouse-liver proteome data and quantification and statistical analysis). For example, the proportion of phosphoproteins is 59.2% for $c_{\text{g}}>3.2\times 10^{4}$ copy number per cell·h^−1^, that is, 25.8% higher than for $c_{\text{g}}<31.8$ copy number per cell·h^−1^ (Figure 6C). This result is partially contributed to by relatively large abundance levels of those phosphoproteins that enhance their overall $c_{\text{g}}$ values (STAR Methods, analysis of Arabidopsis and mouse-liver proteome data). Still, strictly controlling for the abundance ranges of both phospho- and non-phosphoproteins leads to the enrichment of the phosphoproteins with relatively high $c_{\text{g}}$ values, although weak (Figure 6C inset and P=0.04; STAR Methods, analysis of Arabidopsis and mouse-liver proteome data and quantification and statistical analysis). These results indicate that phosphorylation is positively associated not only with the absolute abundance level of a circadian protein, but also with the strength of its oscillation (i.e., the maximum value of $-x^{\prime }\left(t\right)/x\left(t\right)$ in $c_{\text{g}}$). In other words, all the individual elements in the calculation of $c_{\text{g}}$ collectively contribute to the observed tendency between $c_{\text{g}}$ and the circadian phosphoproteins. Together, our proteome-wide analysis indicates a positive association between phosphorylation and the potential biological need for proteosynthetic cost saving represented by $c_{\text{g}}$.

## Discussion

Through quantitative mathematical formulation, we here proposed that the rhythmic degradation rate of proteins with circadian production does not necessarily require rhythmic post-translational regulation. The rhythmic degradation rate spontaneously emerges when the activity of steady-state proteolytic mediators is limited. This rhythmicity can be further amplified by the presence of additional PTMs in the proteolytic pathway. Our theory accounts for empirical patterns of animal and plant core clock proteins as well as those in the proteome-wide data. This work presents an underexplored, generic mechanism of the rhythmic turnover of circadian proteins in a biochemically complementary manner to the previously characterized, explicitly circadian- or diurnal-controlled proteolytic pathways (Cha et al., 2017; Kiba et al., 2007; Lamia et al., 2009; Toledo et al., 2018). We propose that such rhythmic degradation is serving as a plausible solution for relieving circadian biosynthetic costs and thus affording sustained circadian rhythms in many different organisms. Furthermore, the very generic nature of our proposed mechanism indicates potentially more widespread, time-dependent protein turnover in circadian and other contexts than previously thought (Jo et al., 2018; Kiba et al., 2007; Lück et al., 2014; van Ooijen et al., 2011). On the other hand, for any protein with multiple likely mechanisms of rhythmic degradation, our proposed mechanism can be viewed as a null model to examine the maximum *extent* to which the rhythmic degradation is spontaneously developable (as indicated by the largest S values in Figures 5E and 5F), rather than entirely exclude the other sources of the degradation rhythmicity.

The close biological association between circadian clock proteins and protein phosphorylation has been well documented (Farré and Kay, 2007; Reischl and Kramer, 2011; Wang et al., 2010; Zhou et al., 2015), and two-thirds of *Arabidopsis* clock proteins are known phosphoproteins, as discussed above. Our work suggests that another value of this chrono-phospho partnership is to present a natural route to biosynthetic cost saving through rhythmic protein degradation. The proposed mechanism here does not rely on abundance oscillations of the proteolytic mediators, which themselves force additional circadian proteosynthesis that may perhaps compromise biosynthetic cost savings. On the other hand, ATP hydrolysis associated with the proposed phospho-dependent degradation is not a likely factor that undermines the energetic benefit of the biosynthetic cost saving, as we calculated above.

Follow-up expansion of our theoretical framework, in concert with more extensive experimental studies on time-specific protein degradation, is warranted for a comprehensive understanding of circadian turnover dynamics and phenotypic implications (Wirianto et al., 2020; Zhou et al., 2015). A recent report suggests that about one thousand human genes with circadian expression encode the targets, transporters, and metabolizing enzymes of drugs (Ruben et al., 2018). If possible, a massive experimental profiling of drug-related protein turnover over the course of a day would open new avenues to augment our theory in the context of circadian medicine (Panda, 2019). Beyond the scope of the present work, turnover of mRNA or other biomolecules would be analyzable using a similar approach to ours. We also envisage the possibility that a time-of-day-specific translation rate per mRNA molecule (Caster et al., 2016; Lipton et al., 2015) might not be entirely attributed to the circadian control of the translation rate, analogous to our present results on the spontaneous generation of degradation rhythmicity.

### Limitations of the study

Despite the apparent generality of our mathematical framework, its explicit expansion to other processes such as cell cycle and ultradian events may require more comprehensive formulation of energetic demands in molecular economy. On the other hand, our current model includes protein degradation promoted by phosphorylation, but in future versions phosphorylation-inhibited degradation (Hunter, 2007; Yin et al., 2006) needs to be considered as well. In addition, the continuum between our proposed mechanism of spontaneous rhythmic degradation and the conventional degradation mechanisms with circadian or diurnal control remains to be explored.

Although the existing animal and plant data are supportive of our theoretical predictions, experimental tests are clearly warranted including direct validation of our proposed mechanism. This validation could start by identifying (or constructing) a system where a protein with rhythmic expression does not involve either rhythmically controlled degradation pathways or the oscillation of the protein's subcellular partitioning ratio; this system can then be used for testing the presence of rhythmic degradation rates in our predicted conditions. In this sense, the use of appropriately designed synthetic oscillators (Chen et al., 2015; O'Keeffe et al., 2017) may be one avenue for such experimental validation. Although less straight, a more feasible short-term experimental test would be just to measure the degradation rate and/or relevant molecular quantities (e.g., ubiquitinated proportion) of a known circadian protein over the *whole course* of a day with high temporal resolution. One can compare the temporal profile of these data points to our model predictions, provided that the degradation pathway of this protein is not known to involve rhythmic post-translational regulation. Any discrepancy between experimental and theoretical results shall be useful for our model improvement. Consideration of protein localization and stochastic fluctuation (Beesley et al., 2020; Ghim and Almaas, 2009; Kim and Price, 2010) would be necessary for more complete modeling in this subject.

## STAR★Methods

### Key resources table

**Table:** 

REAGENT or RESOURCE	SOURCE	IDENTIFIER
**Software and algorithms**		

Custom codes for model simulation	This paper	https://github.com/rokt-lim/rhythmic-degradation-of-circadian-proteins
Python 3.7.2 & 3.7.4	Python Software Foundation	https://www.python.org
SciPy v1.3.1	Virtanen et al., 2020	https://www.scipy.org

### Resource availability

#### Lead contact

Further information and requests for resources should be directed to and will be fulfilled by the Lead Contact, Pan-Jun Kim (panjunkim@hkbu.edu.hk).

#### Materials availability

This study did not generate any unique reagents.

#### Data and code availability

• This study did not generate new experimental data.
• Source codes for our model simulation have been deposited to the public repository GitHub, and the link is provided in the key resources table.
• Any additional information required to reanalyze the data reported in this paper is available from the lead contact upon request.

### Method details

#### Relationship between protein abundance profiles and biosynthetic costs

We here overview the relationship between waveforms of protein concentrations over time and the cost of protein synthesis in our previous study (Jo et al., 2018). In a circadian system, the protein concentration profile x(t) over time t can often be described by the following equation (Figure 1A):

(Equation 5) $$\frac{dx\left(t\right)}{dt}=g\left(t\right)-r\left(t\right)x\left(t\right),$$

where g(t) and r(t) denote protein synthesis and degradation rates, respectively. Here, we do not assume any specific forms of g(t) and r(t) at the beginning, and they are allowed to take very general forms (e.g., even the functions of any variables such as x(t)). An oscillatory waveform of x(t) satisfies a relation x(t)=x(t+T) where T is an oscillation period. Using the notations ${<g\left(t\right)>}_{t}\equiv \frac{1}{T}\int_{0}^{T}g\left(t\right)dt$ and ${<r\left(t\right)x\left(t\right)>}_{t}\equiv \frac{1}{T}\int_{0}^{T}r\left(t\right)x\left(t\right)dt$, Equation 5 and x(t)=x(t+T) give rise to ${<g\left(t\right)>}_{t}-{<r\left(t\right)x\left(t\right)>}_{t}=\frac{1}{T}\int_{0}^{T}\frac{dx\left(t\right)}{dt}dt=\frac{1}{T}\left[x\left(T\right)-x\left(0\right)\right]=0$. Therefore, ${<g\left(t\right)>}_{t}={<r\left(t\right)x\left(t\right)>}_{t}$. In other words, the periodic nature of the circadian protein levels requires that the proteins must be synthesized as much as they are degraded in each period. The cost of protein synthesis is quantified by the average of the protein amount synthesized per time (Jo et al., 2018), and thus takes the following form for circadian proteins:

$$\frac{\text{Δ}x}{T}=\frac{1}{T}\int_{0}^{T}g\left(t\right)dt={<g\left(t\right)>}_{t}={<r\left(t\right)x\left(t\right)>}_{t}\text{,}$$

where Δx is the protein amount synthesized over the period T. Therefore, given the protein profile x(t) and degradation rate r(t), we calculate the protein synthetic cost ${<r\left(t\right)x\left(t\right)>}_{t}$.

Because g(t)≥0, r(t)≥0, and $g\left(t\right)=x^{\prime }\left(t\right)+r\left(t\right)x\left(t\right)$ ($x^{\prime }\left(t\right)$ is a time derivative of x(t)), we obtain $r\left(t\right)\geq \max\left[-x^{'}\left(t\right)/x\left(t\right),0\right]$ . Hence, the lower bound of the protein degradation rate is determined by the waveform x(t) of the protein profile. This lower bound becomes large for a sharp waveform with a high value of $-x^{\prime }\left(t\right)/x\left(t\right)$ at the falling phase of the oscillation. Protein half-life is equal to $\ln2/r\left(t\right)$, and $-x^{\prime }\left(t\right)/x\left(t\right)$ of some plant and mammalian clock proteins indicates that these proteins have ≲25∼88min-long half-lives at their highest $-x^{\prime }\left(t\right)/x\left(t\right)$ time points (Jo et al., 2018). Establishing such a waveform with high $-x^{\prime }\left(t\right)/x\left(t\right)$ values imposes large values of the lower bound of r(t) and thereby the potentially high protein synthetic cost ${<r\left(t\right)x\left(t\right)>}_{t}$.

If the protein degradation rate is constant over time with r(t)=r, then $r\geq {\text{max}}_{t}\left[-x^{\prime }\left(t\right)/x\left(t\right)\right]$ from the condition $r\geq \max\left[-x^{'}\left(t\right)/x\left(t\right),0\right]$ for arbitrary time t (here, ${\text{max}}_{t}\left[-x^{\prime }\left(t\right)/x\left(t\right)\right]$ denotes the peak value of $-x^{\prime }\left(t\right)/x\left(t\right)$ over time). In other words, a constant degradation rate (i.e., constant half-life) indicates a constantly short-lived protein, which is degraded all the time at least as rapidly as required at the time of the highest $-x^{\prime }\left(t\right)/x\left(t\right)$. The protein synthetic cost satisfies the relation ${<r\cdot x\left(t\right)>}_{t}=r{<x\left(t\right)>}_{t}\geq c_{\text{g}}$ where $c_{\text{g}}\equiv {\text{max}}_{t}\left[-x^{'}\left(t\right)/x\left(t\right)\right]{<x\left(t\right)>}_{t}$. $c_{\text{g}}$ is entirely determined by the waveform x(t) and serves as the lower bound of the cost when the degradation rate is constant. Typically, the sharper is a waveform (i.e. the larger is ${\text{max}}_{t}\left[-x^{\prime }\left(t\right)/x\left(t\right)\right]$), the higher is $c_{\text{g}}$. The circadian clock proteins in our previous study (Jo et al., 2018) exhibit very high values of $c_{\text{g}}$. For example, from $\text{Δ}x\geq c_{\text{g}}T$ using the above $\text{Δ}x/T\geq c_{\text{g}}$, PRR7 and PRR5 proteins must be synthesized per day at least ∼21 and ∼41 times more than actual protein level ${<x\left(t\right)>}_{t}$s, respectively, under the assumption that their degradation rates are constant over time (Jo et al., 2018).

These excessive cost levels can be effectively alleviated by rhythmic degradation rates. If the degradation rate r(t) is not constant over time, r(t) is allowed to become smaller than ${\text{max}}_{t}\left[-x^{\prime }\left(t\right)/x\left(t\right)\right]$ during the time except for the time point of the highest $-x^{\prime }\left(t\right)/x\left(t\right)$. Therefore, the cost can become lower than $c_{\text{g}}$.

#### Biosynthetic cost of mouse liver proteins in the case of constant protein half-lives

We here consider a scenario that protein degradation rates (proportional to the inverse of protein half-lives) are constant over time. Based on the above discussion, the ratio of the biosynthetic cost of proteins with oscillating (circadian or diurnal) abundances to the biosynthetic cost of all detected proteins can be expressed as

$$\frac{\sum_{i\in \text{C}}r_{i}{<x_{i}\left(t\right)>}_{t}}{\sum_{i\in \text{A}}r_{i}{<x_{i}\left(t\right)>}_{t}}\geq \;\frac{\sum_{i\in \text{C}}{\text{max}}_{t}\left[-\frac{x_{i}^{'}\left(t\right)}{x_{i}\left(t\right)}\right]{<x_{i}\left(t\right)>}_{t}}{\sum_{i\in \text{C}}{\text{max}}_{t}\left[-\frac{x_{i}^{'}\left(t\right)}{x_{i}\left(t\right)}\right]{<x_{i}\left(t\right)>}_{t}+\sum_{i\in \left(\text{A}-\text{C}\right)}r_{i}{<x_{i}\left(t\right)>}_{t}}\text{,}$$

where i is an index of each protein, C and A are the sets of proteins with oscillating abundances and all detected proteins, respectively, $x_{i}\left(t\right)$ and $x_{i}^{\prime }\left(t\right)$ denote the concentration of protein i at time t and its derivative, respectively, $r_{i}\;$is a degradation rate of protein i, and ${\left\langle \cdot \right\rangle }_{t}$ and ${\text{max}}_{t}\left[\cdot \right]$ take the average and maximum over time, respectively. In the case of oscillating proteins with constant degradation rates, $r_{i}\geq {\text{max}}_{t}\left[-x_{i}^{\prime }\left(t\right)/x_{i}\left(t\right)\right]$, and this fact leads to the above inequality relation. Here, for a given protein i, ${\text{max}}_{t}\left[-x_{i}^{'}\left(t\right)/x_{i}\left(t\right)\right]{<x_{i}\left(t\right)>}_{t}$ corresponds to $c_{\text{g}}$ defined above. In the case of non-oscillating proteins, we assume that the long-term average level of each protein remains similar over time, and thus the protein production and degradation are balanced in a long term. This long-term balance gives the terms $\sum_{i\in \text{A}}r_{i}{<x_{i}\left(t\right)>}_{t}$ and $\sum_{i\in \left(\text{A}-\text{C}\right)}r_{i}{<x_{i}\left(t\right)>}_{t}$ on the left- and right-hand sides of the above inequality relation, respectively. Here, the value of the right-hand side will be referred to as ${\text{F}}_{\text{cost}}$.

We estimated ${\text{F}}_{\text{cost}}$ for mouse liver proteins: first, we obtained a list of oscillating proteins and their relative abundance profiles (FDR = 0.25) from Data S1 in Mauvoisin et al., 2014. We then obtained the spline curve of the abundance profile of each protein if the protein has at least seven data points across two replicates and more than two pairs of its consecutive data points are not separated with ≥6 hours each. Cubic splines were achieved using scipy.interpolate.interp1d in SciPy v1.3.1, Python 3.7.2, and the results remained similar for other interpolation methods. From the spline curves, we computed the values of ${\text{max}}_{t}\left[-x_{i}^{\prime }\left(t\right)/x_{i}\left(t\right)\right]$. Next, a comprehensive set of detected mouse liver proteins was identified by combining Table S7 in Azimifar et al. (2014) and Data S1 in Mauvoisin et al., 2014. Given the limited availability of data, we used time snapshot data of absolute protein abundances in mouse hepatocyte (Table S7 in Azimifar et al., 2014) as a rough proxy for ${<x_{i}\left(t\right)>}_{t}$ values. Taking the average of protein degradation rates from Data S4 in Karunadharma et al., 2015, we used $r_{i}\approx$ 0.01 h^–1^ in the calculation of $\sum_{i\in \left(\text{A}-\text{C}\right)}r_{i}{<x_{i}\left(t\right)>}_{t}$.

The combination of these data gives rise to $\sum_{i\in \text{C}}{<x_{i}\left(t\right)>}_{t}\approx 1.3\times 10^{8}$ copy number per cell, $\sum_{i\in \text{A}}{<x_{i}\left(t\right)>}_{t}\approx 3.0\times 10^{9}$ copy number per cell, $\sum_{i\in \text{C}}{\text{max}}_{t}\left[-x_{i}^{'}\left(t\right)/x_{i}\left(t\right)\right]{<x_{i}\left(t\right)>}_{t}\approx 8.6\times 10^{6}$ copy number per cell·h^–1^, and $\sum_{i\in \left(\text{A}-\text{C}\right)}r_{i}{<x_{i}\left(t\right)>}_{t}\approx 3.6\times 10^{7}$ copy number per cell·h^–1^. Therefore, $\sum_{i\in \text{C}}{<x_{i}\left(t\right)>}_{t}/\sum_{i\in \text{A}}{<x_{i}\left(t\right)>}_{t}\approx 0.04$ and ${\text{F}}_{\text{cost}}\approx 0.2$. In other words, only ∼4% proteins with oscillating abundances account for at least ∼20% of the total protein synthesis in mouse liver, under the assumption that the protein degradation rates are constant over time.

#### Quantification of degradation rhythmicity

In this study, the rhythmicity of a protein degradation rate r(t) is quantified by $\alpha_{D}\equiv \left\{{\text{max}}_{t}\left[r\left(t\right)\right]-{\text{min}}_{t}\left[r\left(t\right)\right]\right\}/{\text{max}}_{t}\left[r\left(t\right)\right]$, where ${\text{max}}_{t}\left[r\left(t\right)\right]$ and ${\text{min}}_{t}\left[r\left(t\right)\right]$ denote the maximum and minimum values of r(t) over time of day, respectively.

#### Computational modeling of protein ubiquitination without depending on other PTMs

We constructed a model of rhythmically-produced proteins that undergo ubiquitination-mediated degradation, which does not depend on other types of protein modification such as phosphorylation:

(Equation 6) $$\frac{dx_{0}\left(t\right)}{dt}=g\left(t\right)-a_{0}u\left(t\right)x_{0}\left(t\right)+a_{1}x_{E,0}\left(t\right)+sx_{H,ub}\left(t\right),$$

(Equation 7) $$\frac{dx_{E,0}\left(t\right)}{dt}=a_{0}u\left(t\right)x_{0}\left(t\right)-a_{1}x_{E,0}\left(t\right)-qx_{E,0}\left(t\right),$$

(Equation 8) $$\frac{dx_{E,ub}\left(t\right)}{dt}=qx_{E,0}\left(t\right)+a_{0}u\left(t\right)x_{0,ub}\left(t\right)-a_{2}x_{E,ub}\left(t\right)-r_{0}x_{E,ub}\left(t\right),$$

(Equation 9) $$\frac{dx_{0,ub}\left(t\right)}{dt}=a_{2}x_{E,ub}\left(t\right)+b_{1}x_{H,ub}\left(t\right)-b_{0}v\left(t\right)x_{0,ub}\left(t\right)-a_{0}u\left(t\right)x_{0,ub}\left(t\right)-r_{0}x_{0,ub}\left(t\right),$$

(Equation 10) $$\frac{dx_{H,ub}\left(t\right)}{dt}=b_{0}v\left(t\right)x_{0,ub}\left(t\right)-b_{1}x_{H,ub}\left(t\right)-sx_{H,ub}\left(t\right)-r_{0}x_{H,ub}\left(t\right),$$

where t denotes time, g(t) is a protein synthesis rate governed by mRNA-to-protein translation, $x_{0}\left(t\right)$ is the concentration of free proteins without any modifications, $x_{E,0}\left(t\right)$ is the concentration of not-ubiquitinated proteins, binding to ubiquitin ligases, $x_{E,ub}\left(t\right)$ is the concentration of ubiquitinated proteins, binding to ubiquitin ligases, $x_{0,ub}\left(t\right)$ is the concentration of ubiquitinated proteins, not binding to ubiquitin ligases or deubiquitinating enzymes, $x_{H,ub}\left(t\right)$ is the concentration of the ubiquitinated proteins, binding to deubiquitinating enzymes, u(t) is the concentration of ubiquitin ligases, not binding to their substrate proteins, v(t) is the concentration of deubiquitinating enzymes, not binding to their substrate proteins, $r_{0}$ is the degradation rate of ubiquitinated proteins, q is the ubiquitination rate of proteins binding to ubiquitin ligases, s is the deubiquitination rate of proteins binding to deubiquitinating enzymes, lumped with their subsequent dissociation from the deubiquitinating enzymes, $a_{0}$ is the rate of ubiquitin ligase binding to their substrate proteins, $a_{1}$ is the rate of dissociation between ubiquitin ligases and their not-ubiquitinated substrate proteins, $a_{2}$ is the rate of dissociation between ubiquitin ligases and their ubiquitinated proteins, $b_{0}$ is the rate of deubiquitinating enzyme binding to ubiquitinated substrate proteins, and $b_{1}$ is the rate of dissociation between deubiquitinating enzymes and their ubiquitinated substrate proteins.

In the case of circadian or diurnal protein production, g(t)=g(t+T), where T is a period of the protein production. The summation of (Equation 6), (Equation 7), (Equation 8), (Equation 9), (Equation 10) results in the form of Equation 5, when x(t) denotes the total protein concentration as $x\left(t\right)\equiv x_{0}\left(t\right)+x_{E,0}\left(t\right)+x_{E,ub}\left(t\right)+x_{0,ub}\left(t\right)+x_{H,ub}\left(t\right)$ and $r\left(t\right)\equiv r_{0}\left[x_{E,ub}\left(t\right)+x_{0,ub}\left(t\right)+x_{H,ub}\left(t\right)\right]/\left[x_{0}\left(t\right)+x_{E,0}\left(t\right)+x_{E,ub}\left(t\right)+x_{0,ub}\left(t\right)+x_{H,ub}\left(t\right)\right]$. This r(t) is interpreted as the protein degradation rate, regarding its mathematical position in Equation 5. In this study, we assume that ubiquitin ligase and deubiquitinating enzyme concentrations/activities are constant over time, i.e., $\bar{u}\equiv u\left(t\right)+x_{E,0}\left(t\right)+x_{E,ub}\left(t\right)$ and $\bar{v}\equiv v\left(t\right)+x_{H,ub}\left(t\right)$ are constant together with the above parameters not expressed as the functions of t. We then replace u(t) and v(t) in (Equation 6), (Equation 7), (Equation 8), (Equation 9), (Equation 10) by $\bar{u}-x_{E,0}\left(t\right)-x_{E,ub}\left(t\right)$ and $\bar{v}-x_{H,ub}\left(t\right)$, respectively.

For the simulation of our model, we numerically solved (Equation 6), (Equation 7), (Equation 8), (Equation 9), (Equation 10) with given profile g(t) and parameter values, and obtained x(t) and r(t). However, this method makes it difficult to assess the effect of parameter changes on r(t) while controlling for the profile of x(t), because the parameter changes usually affect both r(t) and x(t) together when g(t) is given. Therefore, we implemented another simulation method to maintain the profile of x(t). Specifically, using the relation $x\left(t\right)=x_{0}\left(t\right)+x_{E,0}\left(t\right)+x_{E,ub}\left(t\right)+x_{0,ub}\left(t\right)+x_{H,ub}\left(t\right)$, we replaced $x_{0}\left(t\right)$ in Equation 7 by $x\left(t\right)-x_{E,0}\left(t\right)-x_{E,ub}\left(t\right)-x_{0,ub}\left(t\right)-x_{H,ub}\left(t\right)$, and numerically solved (Equation 7), (Equation 8), (Equation 9), (Equation 10) with given profile x(t) and parameter values. r(t) and g(t) were obtained by $r\left(t\right)=r_{0}\left[x_{E,ub}\left(t\right)+x_{0,ub}\left(t\right)+x_{H,ub}\left(t\right)\right]/x\left(t\right)$ and $g\left(t\right)=x^{\prime }\left(t\right)+r\left(t\right)x\left(t\right)$ from Equation 5, respectively. In this way, we simulated our model with a strictly-maintained profile of x(t) across different parameter values, and g(t) was reversely determined in each of these parameter conditions. The parameter values were selected from physiologically-relevant ranges in Table S1. If the simulation leads any of g(t), $x_{0}\left(t\right)$, u(t), and v(t) to ≤0, we view this combination of the x(t) profile and parameter values as biologically infeasible and abandon its simulation results. For simulation without deubiquitinating enzymes, we set $\bar{v}$ and the initial condition of $x_{H,ub}\left(t\right)$ as zero. For all simulations throughout this study, ordinary differential equations were solved by RK45 or LSODA (scipy.integrate.solve_ivp, rtol = 10^−5^ and atol = 10^−5^) in SciPy v1.3.1 of Python 3.7.4.

We introduce the dimensionless quantities $\tau \equiv r_{0}t,\;X\left(\tau \right)\equiv \frac{a_{0}}{r_{0}}\;\left(\frac{q}{a_{1}+q}\right)x\left(t\right),$ $D\left(\tau \right)\equiv \frac{1}{r_{0}}r\left(t\right)=\left[x_{E,ub}\left(t\right)+x_{0,ub}\left(t\right)+x_{H,ub}\left(t\right)\right]/x\left(t\right)$, $U\equiv \frac{a_{0}}{r_{0}}\;\left(\frac{q}{a_{1}+q}\right)\bar{u}$, and $V\equiv \frac{b_{0}}{r_{0}}\;\left(\frac{r_{0}+s}{b_{1}+r_{0}+s}\right)\bar{v}$. By definition, D(τ)≤1. In the limit of fully ubiquitinated proteins, D(τ)→1 and thus $r\left(t\right)\to r_{0}$. In other words, $r_{0}$ is the theoretical upper limit of r(t). On the other hand, the sum of (Equation 8), (Equation 9), (Equation 10) is equivalent to

(Equation 11) $$\frac{dD\left(\tau \right)x\left(t\right)}{dt}=qx_{E,0}\left(t\right)-sx_{H,ub}\left(t\right)-r_{0}D\left(\tau \right)x\left(t\right)$$

Next, we consider the parameter values that fulfill the following conditions: (*i*) $\text{max}\left(\frac{1}{q},\;\frac{1}{a_{2}},\;\frac{1}{s}\right)\ll T$, (*ii*) $U\ll \frac{q}{r_{0}}$, (*iii*) $V\ll \frac{r_{0}+s}{r_{0}},$ and (*iv*) ${\text{max}}_{\tau }\left[X\left(\tau \right)\right]\ll \text{min}\left[\frac{q}{r_{0}},\;\left(\frac{a_{2}+r_{0}}{r_{0}}\right)\left(\frac{q}{a_{1}+q}\right),\;\frac{a_{0}}{b_{0}}\left(\frac{b_{1}+r_{0}+s}{r_{0}}\right)\left(\frac{q}{a_{1}+q}\right)\right]$. Under the condition (*i*), $x_{E,0}\left(t\right)$, $x_{E,ub}\left(t\right)$, and $x_{H,ub}\left(t\right)$ approximately reach the steady states of Equations 7, 8, and 10 at each time t, respectively. Therefore,

(Equation 12) $$x_{E,0}\left(t\right)\approx \frac{a_{0}u\left(t\right)x_{0}\left(t\right)}{q+a_{1}},$$

(Equation 13) $$x_{E,ub}\left(t\right)\approx \frac{qx_{E,0}\left(t\right)+a_{0}u\left(t\right)x_{0,ub}\left(t\right)}{a_{2}+r_{0}},$$

(Equation 14) $$x_{H,ub}\left(t\right)\approx \frac{b_{0}v\left(t\right)x_{0,ub}\left(t\right)}{s+b_{1}+r_{0}}$$

Under the conditions (*ii*) and (*iii*), Equations 12 and 14 lead to $x_{E,0}\left(t\right)\ll x_{0}\left(t\right)$ and $x_{H,ub}\left(t\right)\ll x_{0,ub}\left(t\right)$, respectively. Under the condition (*iv*), Equation 12 leads to $x_{E,0}\left(t\right)\ll u\left(t\right)$, Equations 12 and 13 lead to $x_{E,ub}\left(t\right)\ll u\left(t\right)$, and Equation 14 leads to $x_{H,ub}\left(t\right)\ll v\left(t\right)$. These results allow the approximation of $x\left(t\right)\approx x_{0}\left(t\right)+x_{E,ub}\left(t\right)+x_{0,ub}\left(t\right)$, $D\left(\tau \right)\approx \left[x_{E,ub}\left(t\right)+x_{0,ub}\left(t\right)\right]/x\left(t\right)$, $\bar{u}\approx u\left(t\right),$ and $\bar{v}\approx v\left(t\right)$. In other words, $x_{0}\left(t\right)\approx \left[1-D\left(\tau \right)\right]x\left(t\right)$ and $x_{E,ub}\left(t\right)\approx D\left(\tau \right)x\left(t\right)-x_{0,ub}\left(t\right)$. Combining these relations with (Equation 12), (Equation 13), (Equation 14) allows one to express $x_{E,0}\left(t\right)$ and $x_{H,ub}\left(t\right)$ as only the functions of D(τ), x(t), $\bar{u}$, and $\bar{v}$. Incorporating these expressions of $x_{E,0}\left(t\right)$ and $x_{H,ub}\left(t\right)$ into Equation 11 gives rise to

(Equation 15) $$\frac{dD\left(\tau \right)X\left(\tau \right)}{d\tau }\approx U\left(1+\frac{BV}{1+AU}\right)\left[1-D\left(\tau \right)\right]X\left(\tau \right)-\left(1+\frac{V}{1+AU}\right)D\left(\tau \right)X\left(\tau \right),$$

where $A\equiv r_{0}\left(a_{1}+q\right)/\left[q\left(a_{2}+r_{0}\right)\right]$ and $B\equiv r_{0}/(a_{2}+r_{0})$. This equation is equivalent to

(Equation 16) $$\frac{dD\left(\tau \right)}{d\tau }\approx \left(1+\frac{BV}{1+AU}\right)U-\left[U+1-R\left(\tau \right)+\frac{1+BU}{1+AU}V\right]D\left(\tau \right),$$

where $R\left(\tau \right)\equiv -X^{\prime }\left(\tau \right)/X\left(\tau \right)$. When V=0 and the change of R(τ) is slow enough for D(τ) to roughly reach a fixed point of Equation 16 at each instant τ (i.e., when the right-hand side of Equation 16 approaches zero for each R(τ)),

(Equation 17) $$D\left(\tau \right)\approx {\left\{1+\frac{1}{U}\left[1-R\left(\tau \right)\right]\right\}}^{-1}$$

This analytical form of D(τ) is equivalent to Equation 1, regarding $D\left(\tau \right)=\frac{1}{r_{0}}r\left(t\right)$ and $\tau =r_{0}t$.

Combining Equation 5 and g(t)≥0 straightforwardly leads to R(τ)≤D(τ)≤1. This relation and Equation 17 give rise to $U≳R_{\text{max}}\equiv {\text{max}}_{\tau }\left[R\left(\tau \right)\right]$. From Equation 17, D(τ) is an approximately increasing function of R(τ), and hence, this D(τ) and r(t) would peak at the time when $R\left(\tau \right)\approx R_{\text{max}}$. Likewise, they would fall into the trough when $R\left(\tau \right)\approx R_{\text{min}}\equiv {\text{min}}_{\tau }\left[R\left(\tau \right)\right]$. Incorporating these results into either Equation 1 or 17 for the calculation of $\alpha_{D}$ leads to Equation 2.

To seek a possible alternative to Equation 17, we assume $\bar{v}=0$ and the following conditions (*v*)–(*vii*) instead of the above (*i*)–(*iv*): (*v*) $\frac{1}{q}\ll T$, (*vi*) ${\text{max}}_{t}\left[x_{E,ub}\left(t\right)/x_{E,0}\left(t\right)\right]$ ≪1, and (*vii*) either ${\text{max}}_{\tau }\left[X\left(\tau \right)\right]\ll U+\frac{q}{r_{0}}$ or $U\ll {\text{min}}_{\tau }\left[X\left(\tau \right)\right]+\frac{q}{r_{0}}$. Under the condition (*v*), $x_{E,0}\left(t\right)$ approximately reaches the steady state of Equation 7 at each time t, and therefore Equation 12 is valid in this case. On the other hand, the condition (*vi*) assures $\bar{u}\approx u\left(t\right)+x_{E,0}\left(t\right)$. Combining this relation with $\left[1-D\left(\tau \right)\right]x\left(t\right)=x_{0}\left(t\right)+x_{E,0}\left(t\right)$ and Equation 12 leads to the approximate form of $x_{E,0}\left(t\right)$ as the function of $\bar{u}$ and [1−D(τ)]x(t), analogous to the exact version of Michaelis–Menten kinetics in Buchler and Louis (2008):

$$x_{E,0}\left(t\right)\approx \frac{1}{2}\left\{\bar{u}+\left[1-D\left(\tau \right)\right]x\left(t\right)+\frac{q+a_{1}}{a_{0}}-\sqrt{{\left\{\bar{u}+\left[1-D\left(\tau \right)\right]x\left(t\right)+\frac{q+a_{1}}{a_{0}}\right\}}^{2}-4\left[1-D\left(\tau \right)\right]\bar{u}x\left(t\right)}\right\}\text{.}$$

Applying the above expression to Equation 11 with $x_{H,ub}\left(t\right)=0$ when $\bar{v}=0$, we obtain

$$\frac{dD\left(\tau \right)}{d\tau }\approx \frac{q}{2r_{0}}\left\{\frac{1}{X\left(\tau \right)}\left(U+\frac{q}{r_{0}}\right)+1-D\left(\tau \right)-\sqrt{{\left[\frac{1}{X\left(\tau \right)}\left(U+\frac{q}{r_{0}}\right)+1-D\left(\tau \right)\right]}^{2}-\frac{4U}{X\left(\tau \right)}\left[1-D\left(\tau \right)\right]}\right\}-\left[1-R\left(\tau \right)\right]D\left(\tau \right)\text{.}$$

We consider the zeroth- and first-order terms of D(τ) in the right-hand side of the above equation, and assume that the changes of X(τ) and R(τ) are slow enough for D(τ) to roughly reach a fixed point at each instant τ (i.e., the right-hand side approaches zero for each X(τ) and R(τ)). Therefore,

$$D\left(\tau \right)\approx \frac{\frac{q}{2r_{0}X\left(\tau \right)}\left\{U+X\left(\tau \right)+\frac{q}{r_{0}}-\sqrt{{\left[U+X\left(\tau \right)+\frac{q}{r_{0}}\right]}^{2}-4UX\left(\tau \right)}\right\}}{\frac{q}{2r_{0}}\left\{1+\frac{U-X\left(\tau \right)-\frac{q}{r_{0}}}{\sqrt{{\left[U+X\left(\tau \right)+\frac{q}{r_{0}}\right]}^{2}-4UX\left(\tau \right)}}\right\}+1-R\left(\tau \right)}\text{.}$$

Because of the condition (*vii*), the above D(τ) is further approximated by

(Equation 18) $$D\left(\tau \right)\approx {\left\{1+\frac{1}{U}\left[1-R\left(\tau \right)\right]\left[1+\frac{r_{0}}{q}U+\frac{r_{0}}{q}X\left(\tau \right)\right]\right\}}^{-1}$$

This analytical form of D(τ) is equivalent to Equation 3, regarding $D\left(\tau \right)=\frac{1}{r_{0}}r\left(t\right)$, $\tau =r_{0}t$, and $X\left(\tau \right)=\frac{a_{0}}{r_{0}}\;\left(\frac{q}{a_{1}+q}\right)x\left(t\right)$. The X(τ) term in Equation 18 originates from Michaelis–Menten kinetics of substrate binding to ubiquitin ligases, but its effect is deflected by the temporal profile of 1−R(τ). Equation 18 is reduced to Equation 17, or equivalently Equation 1, in the case that $U\ll \frac{q}{r_{0}}$ and ${\text{max}}_{\tau }\left[X\left(\tau \right)\right]\ll \frac{q}{r_{0}}$ (similar to the above conditions (*ii*) and (*iv*) in the derivation of Equation 17 and more stringent than the condition (*vii*)).

#### Model application beyond protein ubiquitination

It is clear that our model with (Equation 6), (Equation 7), (Equation 8), (Equation 9), (Equation 10) can be applied for molecular tagging-based degradation mechanisms, mainly protein ubiquitination. Beyond such molecular tagging, we here show that degradation processes triggered by protein complex formation can be approximated by our model. For simplicity, we set V=0 in Equation 15, which is then the same as

$$\frac{dD\left(\tau \right)X\left(\tau \right)}{d\tau }\approx U\left[1-D\left(\tau \right)\right]X\left(\tau \right)-D\left(\tau \right)X\left(\tau \right)$$

If we re-interpret D(τ) as the proportion of a protein that is binding to a proteolytic mediator and thereby committed to degradation, the left-hand side of the equation represents a concentration change of a degradable protein, the first term on the right-hand side stands for complex formation between free protein (∝[1−D(τ)]X(τ)) and proteolytic mediator (∝U), and the second term for protein degradation after the complex formation. The degradation rate is proportional to D(τ). Evidently, the generic form of this equation broadly works for protein degradation triggered by protein complex formation, which does not necessarily involve molecular tagging. For example, in the case of autophagic degradation via interaction of light chain 3 (LC3)-interacting region (LIR) motifs with autophagosome marker LC3 (Birgisdottir et al., 2013; Toledo et al., 2018), this equation can describe a substrate protein sequestered by an LIR-containing protein, or the LIR-containing substrate protein itself targeted for degradation.

#### Computational modeling of phosphorylation-dependent protein ubiquitination

We consider a scenario that protein ubiquitination in a degradation pathway requires n prior phosphorylation events of a substrate protein (n≥1). When n=1 (mono-phosphorylation), the model consists of (Equation 8), (Equation 9), (Equation 10) and the following equations:

(Equation 19) $$\frac{dx_{0}\left(t\right)}{dt}=g\left(t\right)-ky\left(t\right)x_{0}\left(t\right)+lz\left(t\right)x_{p}\left(t\right),$$

(Equation 20) $$\frac{dx_{p}\left(t\right)}{dt}=ky\left(t\right)x_{0}\left(t\right)-lz\left(t\right)x_{p}\left(t\right)-a_{0}u\left(t\right)x_{p}\left(t\right)+a_{1}x_{E,0}\left(t\right)+sx_{H,ub}\left(t\right),$$

(Equation 21) $$\frac{dx_{E,0}\left(t\right)}{dt}=a_{0}u\left(t\right)x_{p}\left(t\right)-a_{1}x_{E,0}\left(t\right)-qx_{E,0}\left(t\right),$$

where $x_{p}\left(t\right)$ is the concentration of phosphorylated but not ubiquitinated proteins, not binding to any enzymes at time t, y(t) is the concentration of protein kinases, z(t) is the concentration of protein phosphatases, k is the rate of kinase binding to substrate proteins, lumped with subsequent phosphorylation and substrate dissociation, and l is the rate of phosphatase binding to phosphorylated substrate proteins, lumped with subsequent dephosphorylation and substrate dissociation.

When n>1 (multisite phosphorylation), the model consists of (Equation 8), (Equation 9), (Equation 10) and the following equations:

(Equation 22) $$\frac{dx_{0}\left(t\right)}{dt}=g\left(t\right)-k_{1}y\left(t\right)x_{0}\left(t\right)+l_{1}z\left(t\right)x_{p_{1}}\left(t\right),$$

(Equation 23) $$\frac{dx_{p_{1}}\left(t\right)}{dt}=k_{1}y\left(t\right)x_{0}\left(t\right)+l_{2}z\left(t\right)x_{p_{2}}\left(t\right)-l_{1}z\left(t\right)x_{p_{1}}\left(t\right)-k_{2}y\left(t\right)x_{p_{1}}\left(t\right),$$

$$\frac{dx_{p_{i}}\left(t\right)}{dt}=k_{i}y\left(t\right)x_{p_{i-1}}\left(t\right)+l_{i+1}z\left(t\right)x_{p_{i+1}}\left(t\right)-l_{i}z\left(t\right)x_{p_{i}}\left(t\right)-k_{i+1}y\left(t\right)x_{p_{i}}\left(t\right)$$

(Equation 24) $$\left(i=2,\;3,\;\cdots ,\;n-1\;for\;n>2\right),$$

(Equation 25) $$\frac{dx_{p_{n}}\left(t\right)}{dt}=k_{n}y\left(t\right)x_{p_{n-1}}\left(t\right)-l_{n}z\left(t\right)x_{p_{n}}\left(t\right)-a_{0}u\left(t\right)x_{p_{n}}\left(t\right)+a_{1}x_{E,0}\left(t\right)+sx_{H,ub}\left(t\right),$$

(Equation 26) $$\frac{dx_{E,0}\left(t\right)}{dt}=a_{0}u\left(t\right)x_{p_{n}}\left(t\right)-a_{1}x_{E,0}\left(t\right)-qx_{E,0}\left(t\right),$$

where $x_{p_{i}}\left(t\right)$ denotes the concentration of the *i*^th^ phosphorylated (but not ubiquitinated) proteins, which are not binding to any enzymes at time t, $k_{i}$ and $l_{i}$ are the extended versions of the parameters k and l in Equations 19 and 20, specific to the (*i* −1)^th^ and *i*^th^ phosphorylated proteins, respectively.

In the same manner as the phospho-independent case, g(t)=g(t+T), where T is a period of protein production. The summation of (Equation 8), (Equation 9), (Equation 10) and either (Equation 19), (Equation 20), (Equation 21) (for n=1) or (Equation 22), (Equation 23), (Equation 24), (Equation 25), (Equation 26) (for n>1) results in the form of Equation 5, when x(t) denotes the total protein concentration as $x\left(t\right)\equiv x_{0}\left(t\right)+x_{p}\left(t\right)+x_{E,0}\left(t\right)+x_{E,ub}\left(t\right)+x_{0,ub}\left(t\right)+x_{H,ub}\left(t\right)$ (n=1) or $x\left(t\right)\equiv x_{0}\left(t\right)+\overset{n}{\underset{i=1}{\sum }}x_{p_{i}}\left(t\right)+x_{E,0}\left(t\right)+x_{E,ub}\left(t\right)+x_{0,ub}\left(t\right)+x_{H,ub}\left(t\right)$ (n>1), and $r\left(t\right)\equiv r_{0}\left[x_{E,ub}\left(t\right)+x_{0,ub}\left(t\right)+x_{H,ub}\left(t\right)\right]/x\left(t\right)$. Similar to the previous case, this r(t) is interpreted as a protein degradation rate, regarding its mathematical position in Equation 5. We assume that kinase, phosphatase, ubiquitin ligase, and deubiquitinating enzyme concentrations/activities are constant over time, i.e., y≡y(t), z≡z(t), $\bar{u}\equiv u\left(t\right)+x_{E,0}\left(t\right)+x_{E,ub}\left(t\right)$, and $\bar{v}\equiv v\left(t\right)+x_{H,ub}\left(t\right)$ are constant together with the above parameters not expressed as the functions of t. We then replace y(t), z(t), u(t), and v(t) in (Equation 8), (Equation 9), (Equation 10) and (Equation 19), (Equation 20), (Equation 21), (Equation 22), (Equation 23), (Equation 24), (Equation 25), (Equation 26) by y, z, $\bar{u}-x_{E,0}\left(t\right)-x_{E,ub}\left(t\right)$, and $\bar{v}-x_{H,ub}\left(t\right)$, respectively.

In a similar fashion to the phospho-independent case, we controlled for the profile of x(t) in our model simulation by replacing $x_{0}\left(t\right)$ in Equations 20 and 23 by $x\left(t\right)-x_{p}\left(t\right)-x_{E,0}\left(t\right)-x_{E,ub}\left(t\right)-x_{0,ub}\left(t\right)-x_{H,ub}\left(t\right)$ and$\;x\left(t\right)-\overset{n}{\underset{i=1}{\sum }}x_{p_{i}}\left(t\right)-x_{E,0}\left(t\right)-x_{E,ub}\left(t\right)-x_{0,ub}\left(t\right)-x_{H,ub}\left(t\right)$, respectively. We numerically solved (Equation 8), (Equation 9), (Equation 10) and either Equations 20 and 21 (for n=1) or (Equation 23), (Equation 24), (Equation 25), (Equation 26) (for n>1) with given profile x(t) and parameter values. r(t) and g(t) were obtained by $r\left(t\right)=r_{0}\left[x_{E,ub}\left(t\right)+x_{0,ub}\left(t\right)+x_{H,ub}\left(t\right)\right]/x\left(t\right)$ and $g\left(t\right)=x^{\prime }\left(t\right)+r\left(t\right)x\left(t\right)$ from Equation 5, respectively. In this way, we simulated our model with a strictly-maintained profile of x(t) across different parameter values and g(t) was reversely determined in each of these parameter conditions. The parameter values were selected from physiologically-relevant ranges in Table S1. Throughout this study, uniform sampling of parameter values was conducted by the Mersenne Twister in random.py of Python 3.7.4. If the simulation of particular parameter values leads any of g(t), $x_{0}\left(t\right)$, u(t), and v(t) to ≤0, we view this combination of the x(t) profile and parameter values as biologically infeasible and abandon its simulation results. For simulation without phosphatases and deubiquitinating enzymes, we set z, $\bar{v}$, and the initial condition of $x_{H,ub}\left(t\right)$ as zero.

In addition to the previous dimensionless quantities τ, X(τ), D(τ), U, and V, we introduce $W\left(\tau \right)\equiv x_{p}\left(t\right)/x\left(t\right)$, $Y\equiv \frac{k}{r_{0}}y$, and $Z\equiv \left(l/r_{0}\right)z$, when n=1. We further consider the parameter values that fulfill the aforementioned conditions (*i*)–(*iv*) in the phospho-independent case. Based on these parameters, we take the pseudo-steady state approximation of the model with n=1 in a similar fashion to the derivation of Equation 16, and then obtain

(Equation 27) $$\frac{dW\left(\tau \right)}{d\tau }\approx Y-\left[Y+U-R\left(\tau \right)+Z+\frac{BQUV}{1+AU}\right]W\left(\tau \right)-\left(Y-\frac{QV}{1+AU}\right)D\left(\tau \right),$$

(Equation 28) $$\frac{dD\left(\tau \right)}{d\tau }\approx U\left(1+\frac{BV}{1+AU}\right)W\left(\tau \right)-\left[1-R\left(\tau \right)+\frac{V}{1+AU}\right]D\left(\tau \right),$$

where R(τ), A, and B are the same as those in Equation 16 and $Q\equiv s/(r_{0}+s)$. When Z=V=0 and the change of R(τ) is slow enough forW(τ) and D(τ) to roughly reach a fixed point of Equations 27 and 28 at each instant τ,

(Equation 29) $$D\left(\tau \right)\approx {\left\{\left\{1+\frac{1}{U}\left[1-R\left(\tau \right)\right]\right\}\left[1-\frac{1}{Y}R\left(\tau \right)\right]+\frac{1}{Y}\right\}}^{-1}$$

This analytical form of D(τ) is equivalent to Equation 4, regarding $D\left(\tau \right)=\frac{1}{r_{0}}r\left(t\right)$ and $\tau =r_{0}t$. Combining Equation 5 and g(t)≥0 straightforwardly leads to R(τ)≤D(τ)≤1. This relation and Equation 29 give rise to $\;U≳R_{\text{max}}\equiv {\text{max}}_{\tau }\left[R\left(\tau \right)\right]$.

Following a similar procedure to the derivation of Equation 29, the pseudo-steady state approximation of the model with arbitrary n for multisite phosphorylation leads to

$$D\left(\tau \right)\approx \frac{UK_{1}K_{2}\cdots K_{n}Y^{n}}{\eta_{n}\left(R_{\tau }\right)},$$

where $K_{i}\equiv \frac{k_{i}}{k_{1}}$ (i=1,2,⋯,n), $R_{\tau }\equiv R\left(\tau \right)$, and $\eta_{n}\left(R_{\tau }\right)\equiv UK_{1}K_{2}\cdots K_{n}Y^{n}+K_{1}K_{2}\cdots K_{n}Y^{n}\left(1-R_{\tau }\right)+K_{1}K_{2}\cdots K_{n-1}Y^{n-1}\left(1-R_{\tau }\right)\left(U-R_{\tau }\right)+K_{1}K_{2}\cdots K_{n-2}Y^{n-2}\left(1-R_{\tau }\right)\left(U-R_{\tau }\right)\left(K_{n}Y-R_{\tau }\right)+K_{1}K_{2}\cdots K_{n-3}Y^{n-3}\left(1-R_{\tau }\right)\left(U-R_{\tau }\right)\left(K_{n}Y-R_{\tau }\right)\left(K_{n-1}Y-R_{\tau }\right)+\cdots +K_{1}K_{2}Y^{2}\left(1-R_{\tau }\right)\left(U-R_{\tau }\right)\prod_{i=4}^{n}\left(K_{i}Y-R_{\tau }\right)+\left(1-R_{\tau }\right)\left(U-R_{\tau }\right)\left(K_{1}Y+K_{2}Y-R_{\tau }\right)\prod_{i=3}^{n}\left(K_{i}Y-R_{\tau }\right)$.

The above D(τ) is inversely proportional to $\eta_{n}\left(R_{\tau }\right)/(K_{1}K_{2}\cdots K_{n}Y^{n})$. From the above form of $\eta_{n}\left(R_{\tau }\right)$, the explicit calculation of $\eta_{n}\left(R_{\tau }\right)/(K_{1}K_{2}\cdots K_{n}Y^{n})$ shows the repetitive presence of a term $\left[1-R_{\tau }/(K_{i}Y)\right]$. The temporal variation of this term, driven by the oscillation of $R_{\tau }$, is particularly large when $K_{i}Y$ is small. In other words, among the kinase binding rates across multiple phosphosites, the lowest binding rates ($\propto \min_{i}\left(K_{i}\right)$) determine the overall rhythmicity of a degradation rate (∝D(τ)). These limiting steps of phosphorylation also constrain the overall cost of protein production, because the cost is proportional to ${<D\left(\tau \right)X\left(\tau \right)>}_{\tau }$ and thus strongly affected by the rhythmicity of D(τ).

#### Model application for PTMs beyond phosphorylation

Our model with (Equation 8), (Equation 9), (Equation 10) and (Equation 19), (Equation 20), (Equation 21), (Equation 22), (Equation 23), (Equation 24), (Equation 25), (Equation 26) is based on ubiquitination promoted by phosphorylation, but other types of PTMs as targeting signals for ubiquitination can also be addressed by this model. For simplicity, we set Z=V=0 in Equations 27 and 28, which are then equivalent to

$$\frac{dW\left(\tau \right)X\left(\tau \right)}{d\tau }\approx Y\left[1-W\left(\tau \right)-D\left(\tau \right)\right]X\left(\tau \right)-UW\left(\tau \right)X\left(\tau \right)\text{,}$$

$$\frac{dD\left(\tau \right)X\left(\tau \right)}{d\tau }\approx UW\left(\tau \right)X\left(\tau \right)-D\left(\tau \right)X\left(\tau \right)\text{.}$$

If we re-interpret W(τ) as the proportion of a protein that is modified in some ways for subsequent ubiquitination, the left-hand sides of the first and second equations represent concentration changes of this modified and the ubiquitinated proteins, respectively. In the first equation, the first term on the right-hand side stands for the enzymatic modification (∝Y) of an unmodified substrate (∝[1−W(τ)−D(τ)]X(τ)), and the second term for the ubiquitination (∝U(τ)) of the modified substrate (∝W(τ)X(τ)). In the second equation, the degradation rate is proportional to a ubiquitinated fraction (D(τ)) on the right-hand side. Clearly, the generic forms of these equations work for the modification types that do not necessarily involve phosphorylation. The examples include SUMOylation (Miteva et al., 2010) and PTMs in the N-degron pathway (Dissmeyer, 2019).

#### Model expansion for multiple degradation routes

We expanded (Equation 8), (Equation 9), (Equation 10) and (Equation 22), (Equation 23), (Equation 24), (Equation 25), (Equation 26) with n=4 to a scenario of protein degradation where more than one degradation route exists for a given protein with multiple phosphorylation events. The model consists of Equation 22 and the following equations:

$$\frac{dx_{p_{1}}\left(t\right)}{dt}=k_{1}y\left(t\right)x_{0}\left(t\right)+l_{2}z\left(t\right)x_{p_{2}}\left(t\right)-l_{1}z\left(t\right)x_{p_{1}}\left(t\right)-k_{2}y\left(t\right)x_{p_{1}}\left(t\right)-a_{1,0}u_{1}\left(t\right)x_{p_{1}}\left(t\right)+a_{1,1}x_{1,E,0}\left(t\right)+s_{1}x_{1,H,ub}\left(t\right),$$

$$\frac{dx_{p_{2}}\left(t\right)}{dt}=k_{2}y\left(t\right)x_{p_{1}}\left(t\right)+l_{3}z\left(t\right)x_{p_{3}}\left(t\right)-l_{2}z\left(t\right)x_{p_{2}}\left(t\right)-k_{3}y\left(t\right)x_{p_{2}}\left(t\right)\;-a_{2,0}u_{2}\left(t\right)x_{p_{2}}\left(t\right)+a_{2,1}x_{2,E,0}\left(t\right)+s_{2}x_{2,H,ub}\left(t\right),\;$$

$$\frac{dx_{p_{3}}\left(t\right)}{dt}=k_{3}y\left(t\right)x_{p_{2}}\left(t\right)+l_{4}z\left(t\right)x_{p_{4}}\left(t\right)-l_{3}z\left(t\right)x_{p_{3}}\left(t\right)-k_{4}y\left(t\right)x_{p_{3}}\left(t\right)-a_{3,0}u_{3}\left(t\right)x_{p_{3}}\left(t\right)+a_{3,1}x_{3,E,0}\left(t\right)+s_{3}x_{3,H,ub}\left(t\right),$$

$$\frac{dx_{p_{4}}\left(t\right)}{dt}=k_{4}y\left(t\right)x_{p_{3}}\left(t\right)-l_{4}z\left(t\right)x_{p_{4}}\left(t\right)-a_{4,0}u_{4}\left(t\right)x_{p_{4}}\left(t\right)+a_{4,1}x_{4,E,0}\left(t\right)+s_{4}x_{4,H,ub}\left(t\right),$$

$$\frac{dx_{i,E,0}\left(t\right)}{dt}=a_{i,0}u_{i}\left(t\right)x_{p_{i}}\left(t\right)-a_{i,1}x_{i,E,0}\left(t\right)-q_{i}x_{i,E,0}\left(t\right)\;\left(i=1,\;2,\;3,\;4\right),$$

$$\frac{dx_{i,E,ub}\left(t\right)}{dt}=q_{i}x_{i,E,0}\left(t\right)+a_{i,0}u_{i}\left(t\right)x_{i,0,ub}\left(t\right)-a_{i,2}x_{i,E,ub}\left(t\right)-r_{i,0}x_{i,E,ub}\left(t\right)\;\left(i=1,\;2,\;3,\;4\right)\text{,}$$

$$\frac{dx_{i,0,ub}\left(t\right)}{dt}=a_{i,2}x_{i,E,ub}\left(t\right)+b_{i,1}x_{i,H,ub}\left(t\right)-b_{i,0}v_{i}\left(t\right)x_{i,0,ub}\left(t\right)-a_{i,0}u_{i}\left(t\right)x_{i,0,ub}\left(t\right)-r_{i,0}x_{i,0,ub}\left(t\right)\;\left(i=1,\;2,\;3,\;4\right),$$

$$\frac{dx_{i,H,ub}\left(t\right)}{dt}=b_{i,0}v_{i}\left(t\right)x_{i,0,ub}\left(t\right)-b_{i,1}x_{i,H,ub}\left(t\right)-s_{i}x_{i,H,ub}\left(t\right)-r_{i,0}x_{i,H,ub}\left(t\right)\;\left(i=1,\;2,\;3,\;4\right),$$

where the variables and parameters are the same as in (Equation 8), (Equation 9), (Equation 10) and (Equation 23), (Equation 24), (Equation 25), (Equation 26), except those in ubiquitination/deubiquitination of phosphorylated proteins. Specifically, $x_{i,E,0}\left(t\right)$, $x_{i,E,ub}\left(t\right)$, $x_{i,0,ub}\left(t\right)$, $x_{i,H,ub}\left(t\right)$, and $r_{i,0}$ are the counterparts of $x_{E,0}\left(t\right)$, $x_{E,ub}\left(t\right)$, $x_{0,ub}\left(t\right)$, $x_{H,ub}\left(t\right)$, and $r_{0}$ in (Equation 8), (Equation 9), (Equation 10), 25, and 26 for the *i*^th^ phosphorylated protein. $u_{i}\left(t\right)$ and $v_{i}\left(t\right)$ denote the concentrations of free ubiquitin ligase and deubiquitinating enzyme specific to the *i*^th^ phosphorylated protein, respectively. $a_{i,0}$, $a_{i,1}$, $a_{i,2}$, and $q_{i}$ are the counterparts of $a_{0}$, $a_{1}$, $a_{2}$, and q in (Equation 8), (Equation 9), (Equation 10), 25, and 26 for the ubiquitin ligase specific to the *i*^th^ phosphorylated protein. $b_{i,0}$, $b_{i,1}$, and $s_{i}$ are the counterparts of $b_{0}$, $b_{1}$, and s in (Equation 8), (Equation 9), (Equation 10), 25, and 26 for the deubiquitinating enzyme specific to the *i*^th^ phosphorylated protein.

We assume that kinase, phosphatase, ubiquitin ligase, and deubiquitinating enzyme levels/activities are constant over time, i.e., ${\bar{u}}_{i}\equiv u_{i}\left(t\right)+x_{i,E,0}\left(t\right)+x_{i,E,ub}\left(t\right)$, ${\bar{v}}_{i}\equiv v_{i}\left(t\right)+x_{i,H,ub}\left(t\right)$, y≡y(t), and z≡z(t) are constant together with the above parameters not expressed as the functions of t. In a similar way to the previous cases, we performed numerical simulations of this model and obtained the protein degradation rate, its rhythmicity $\alpha_{D}$, and the protein synthetic cost (Figure S2). We simulated different combinations of degradation routes by setting the ubiquitin ligase levels (${\bar{u}}_{i}$) in irrelevant degradation pathways as zero, and otherwise setting ${\bar{u}}_{i}>0$. The specific parameter values were chosen from physiologically-relevant ranges in Table S1.

#### Known phosphoproteins in the *Arabidopsis* circadian clock

We checked whether the following clock proteins in plant *Arabidopsis thaliana* are listed as phosphoproteins in the *Arabidopsis* Protein Phosphorylation Site Database (*PhosPhAt*) 4.0 (Durek et al., 2010): LATE ELONGATED HYPOCOTYL (LHY), CIRCADIAN CLOCK ASSOCIATED 1 (CCA1), PRR9, PRR7, PRR5, TIMING OF CAB EXPRESSION 1 (TOC1), EARLY FLOWERING 3, 4 (ELF3, ELF4), LUX ARRHYTHMO (LUX), GIGANTEA (GI ), ZEITLUPE (ZTL), and REVEILLE 8 (RVE8).

Among these proteins, LHY, CCA1, PRR7, PRR5, ELF4, LUX, GI , and RVE8 were classified as phosphoproteins with the cited experimental evidence in *PhosPhAt* 4.0.

#### Analysis of TIM ubiquitination data

We collected the circadian profiles of the TIM's abundance and ubiquitinated fraction in constant darkness in Szabó et al., 2018. Specifically, two replicates of the TIM abundance levels associated with Figure 3A of Szabó et al., 2018 were kindly provided by Áron Szabó, and normalized by their average at CT 18 hr. To achieve the protein profile x(t) (Figure 4A), we obtained the smoothing cubic spline of the average abundances with k = 3 (degree of the spline fit) and s = 5 (smoothing condition) using scipy.interpolate.splrep in SciPy v1.3.1, Python 3.7.2. This profile x(t) was used for the calculation of $-x^{\prime }\left(t\right)/x\left(t\right)$ (Figure 4B). The average and standard deviation of the proportions of ubiquitinated TIM at each time point were retrieved from Figure 3B of Szabó et al., 2018. In this literature source, the average proportion of ubiquitinated TIM at CT 0 hr was scaled to 100, and we rescaled it to 1 (Figure 4B).

#### PRR7 and PER2 degradation modeling and analysis

To address the cases of PRR7 and PER2 degradation, we adopted the phospho-independent and phospho-dependent protein degradation models in (Equation 6), (Equation 7), (Equation 8), (Equation 9), (Equation 10) and (Equation 19), (Equation 20), (Equation 21), (Equation 22), (Equation 23), (Equation 24), (Equation 25), (Equation 26). The protein kinase, phosphatase, ubiquitin ligase, and deubiquitinating enzyme levels/activities were assumed to be constant over time. Following the aforementioned procedure of our model simulation, we maintained the profile of x(t) set to the experimental PRR7 or PER2 abundance profile (Figures 5A and 5B) and obtained the ensemble of r(t) with uniformly-sampled parameter sets in physiologically-relevant ranges in Table S1. For the experimental PRR7 abundance profile, we obtained the abundance data with 2-hour resolution under equal length light-dark cycles in Figure 5A of Nakamichi et al., 2010 and used the cubic spline curve. For the experimental PER2 abundance profile, we obtained the abundance data with 0.1-hour resolution (cycloheximide-untreated control data) in Figure 1A of Zhou et al. (2015) and smoothened them with a moving window average (3-hour window).

For each simulated r(t), we computed the protein synthetic cost ${<r\left(t\right)x\left(t\right)>}_{t}$ as derived above. In addition, we measured similarity S between the simulated r(t) and the empirical degradation-rate profile $r^{\text{E}}\left(t\right)$, as follows:

$$S\equiv \frac{\int_{0}^{T}\text{min}\left[r\left(t\right),\;r^{\text{E}}\left(t\right)\right]dt}{\int_{0}^{T}\text{max}\left[r\left(t\right),\;r^{\text{E}}\left(t\right)\right]dt}\text{,}$$

where T is an oscillation period of r(t) and $r^{\text{E}}\left(t\right)$. S takes a range of 0≤S≤1, and becomes large for quantitatively similar trajectories of r(t) and $r^{\text{E}}\left(t\right)$. In this study, the alternative definition of S did not much change our results. Given the scarcity of the experimental PRR7 and PER2 degradation rates, we estimated their $r^{\text{E}}\left(t\right)$ profiles in the following way: first, we obtained the experimental PRR7 degradation rates at zeitgeber time 4 hr, 12 hr, and 18 hr (Farré and Kay, 2007) and the experimental PER2 degradation rates at t= 19 hr, 22 hr, 25 hr, 28 hr, and 30 hr (Zhou et al., 2015), as detailed in Jo et al. (2018). Because $r^{\text{E}}\left(t\right)$ is required to satisfy $r^{\text{E}}\left(t\right)\geq \max\left[-x^{'}\left(t\right)/x\left(t\right),0\right]$ as shown above, the linear inter- or extrapolation of the experimental degradation rates was compared with $\max\left[-x^{'}\left(t\right)/x\left(t\right),0\right]$ at each time t, and a higher value between them was chosen for the estimation of $r^{\text{E}}\left(t\right)$. In the case of PER2, $-x^{\prime }\left(t\right)/x\left(t\right)$ derived from x(t) was very noisy, and therefore smoothened with a moving window average (1-hour window). For PRR7 or PER2, we set the lower bound of $r^{\text{E}}\left(t\right)$ to the minimum value of the experimental degradation rates. The inferred profiles of $r^{\text{E}}\left(t\right)$ are presented in Figures 5A and 5B.

In the simulation conditions in Figure 5, the phosphatase and deubiquitinating enzyme levels were set to zero. In addition, $k_{1}$, $k_{2}$, ···, $k_{n}$ in (Equation 21), (Equation 22), (Equation 23), (Equation 24) were set to the identical values. We tried this simplification because the lowest kinase binding rate ($\propto min\left(k_{1},\;k_{2},\cdots ,\;k_{n}\right)$) in multisite phosphorylation governs the substrate's overall degradation dynamics (Figures 3D and S4) and therefore unifying the kinase binding rates would reduce the model complexity without disrupting the major mode of the degradation dynamics. Nevertheless, the PRR7 simulation without those constraints, as well as the simulation of our full realistic model of the PER2 degradation, did not much affect the main results (Figure S5). Here, the realistic PER2 degradation model was modified from the previous model in Zhou et al. (2015) and comprised the following equations:

$$\frac{dx_{0}\left(t\right)}{dt}=g\left(t\right)-k_{1}y\left(t\right)x_{0}\left(t\right)+l_{1}z\left(t\right)x_{p_{1}}\left(t\right)-k_{\text{β}}y\left(t\right)x_{0}\left(t\right)+l_{\text{β}}z\left(t\right)x_{p_{\text{β}}}\left(t\right)-a_{0,0}u\left(t\right)x_{0}\left(t\right)+a_{0,1}x_{0,E,0}\left(t\right)+s_{0}x_{0,H,ub}\left(t\right),$$

$$\frac{dx_{p_{1}}\left(t\right)}{dt}=k_{1}y\left(t\right)x_{0}\left(t\right)+l_{2}z\left(t\right)x_{p_{2}}\left(t\right)-l_{1}z\left(t\right)x_{p_{1}}\left(t\right)-k_{2}y\left(t\right)x_{p_{1}}\left(t\right)-a_{1,0}u\left(t\right)x_{p_{1}}\left(t\right)+a_{1,1}x_{1,E,0}\left(t\right)+s_{1}x_{1,H,ub}\left(t\right),$$

$$\frac{dx_{p_{2}}\left(t\right)}{dt}=k_{2}y\left(t\right)x_{p_{1}}\left(t\right)+l_{3}z\left(t\right)x_{p_{3}}\left(t\right)-l_{2}z\left(t\right)x_{p_{2}}\left(t\right)-k_{3}y\left(t\right)x_{p_{2}}\left(t\right)\;-a_{2,0}u\left(t\right)x_{p_{2}}\left(t\right)+a_{2,1}x_{2,E,0}\left(t\right)+s_{2}x_{2,H,ub}\left(t\right),\;$$

$$\frac{dx_{p_{3}}\left(t\right)}{dt}=k_{3}y\left(t\right)x_{p_{2}}\left(t\right)+l_{4}z\left(t\right)x_{p_{4}}\left(t\right)-l_{3}z\left(t\right)x_{p_{3}}\left(t\right)-k_{4}y\left(t\right)x_{p_{3}}\left(t\right)-a_{3,0}u\left(t\right)x_{p_{3}}\left(t\right)+a_{3,1}x_{3,E,0}\left(t\right)+s_{3}x_{3,H,ub}\left(t\right),$$

$$\frac{dx_{p_{4}}\left(t\right)}{dt}=-l_{4}z\left(t\right)x_{p_{4}}\left(t\right)+k_{4}y\left(t\right)x_{p_{3}}\left(t\right)-a_{4,0}u\left(t\right)x_{p_{4}}\left(t\right)+a_{4,1}x_{4,E,0}\left(t\right)+s_{4}x_{4,H,ub}\left(t\right),$$

$$\frac{dx_{i,E,0}\left(t\right)}{dt}=a_{i,0}u\left(t\right)x_{p_{i}}\left(t\right)-a_{i,1}x_{E,0}\left(t\right)-q_{i}x_{i,E,0}\left(t\right)\;\left[i=0,\;1,\;2,\;3,\;4;\;x_{p_{0}}\left(t\right)\equiv x_{0}\left(t\right)\right],$$

$$\frac{dx_{i,E,ub}\left(t\right)}{dt}=q_{i}x_{i,E,0}\left(t\right)+a_{i,0}u\left(t\right)x_{i,0,ub}\left(t\right)-a_{i,2}x_{i,E,ub}\left(t\right)-r_{i,0}x_{i,E,ub}\left(t\right)\;\left(i=0,\;1,\;2,\;3,\;4\right),$$

$$\frac{dx_{i,0,ub}\left(t\right)}{dt}=a_{i,2}x_{i,E,ub}\left(t\right)+b_{i,1}x_{i,H,ub}\left(t\right)-b_{i,0}v\left(t\right)x_{i,0,ub}\left(t\right)-a_{i,0}u\left(t\right)x_{i,0,ub}\left(t\right)-r_{i,0}x_{i,0,ub}\left(t\right)\;\left(i=0,\;1,\;2,\;3,\;4\right)$$

$$\frac{dx_{i,H,ub}\left(t\right)}{dt}=b_{i,0}v\left(t\right)x_{i,0,ub}\left(t\right)-b_{i,1}x_{i,H,ub}\left(t\right)-s_{i}x_{i,H,ub}\left(t\right)-r_{i,0}x_{i,H,ub}\left(t\right)\;\left(i=0,\;1,\;2,\;3,\;4\right),$$

$$\frac{dx_{p_{\text{β}}}\left(t\right)}{dt}=-l_{\text{β}}z\left(t\right)x_{p_{\text{β}}}\left(t\right)+k_{\text{β}}y\left(t\right)x_{0}\left(t\right)-a_{\text{β},0}u_{\text{β}}\left(t\right)x_{p_{\text{β}}}\left(t\right)+a_{\text{β},1}x_{\text{β},E,0}\left(t\right)+s_{\text{β}}x_{\text{β},H,ub}\left(t\right),$$

$$\frac{dx_{\text{β},E,0}\left(t\right)}{dt}=a_{\text{β},0}u_{\text{β}}\left(t\right)x_{p_{\text{β}}}\left(t\right)-a_{\text{β},1}x_{\text{β},E,0}\left(t\right)-q_{\text{β}}x_{\text{β},E,0}\left(t\right),$$

$$\frac{dx_{\text{β},E,ub}\left(t\right)}{dt}=q_{\text{β}}x_{\text{β},\;E,0}\left(t\right)+a_{\text{β},0}u_{\text{β}}\left(t\right)x_{\text{β},0,ub}\left(t\right)-a_{\text{β},2}x_{\text{β},E,ub}\left(t\right)-r_{\text{β},0}x_{\text{β},E,ub}\left(t\right),$$

$$\frac{dx_{\text{β},0,ub}\left(t\right)}{dt}=a_{\text{β},2}x_{\text{β},E,ub}\left(t\right)+b_{\text{β},1}x_{\text{β},H,ub}\left(t\right)-b_{\text{β},0}v_{\text{β}}\left(t\right)x_{\text{β},0,ub}\left(t\right)-a_{\text{β},0}u_{\text{β}}\left(t\right)x_{\text{β},0,ub}\left(t\right)-r_{\text{β},0}x_{\text{β},0,ub}\left(t\right),$$

$$\frac{dx_{\text{β},H,ub}\left(t\right)}{dt}=b_{\text{β},0}v_{\text{β}}\left(t\right)x_{\text{β},0,ub}\left(t\right)-b_{\text{β},1}x_{\text{β},H,ub}\left(t\right)-s_{\text{β}}x_{\text{β},H,ub}\left(t\right)-r_{\text{β},0}x_{\text{β},H,ub}\left(t\right).$$

The above variables and parameters are the overall same as in the model with multisite phosphorylation-dependent degradation ((Equation 8), (Equation 9), (Equation 10) and (Equation 22), (Equation 23), (Equation 24), (Equation 25), (Equation 26)). To be clear, subscripts i and $p_{i}$ correspond to the *i*^th^ phosphorylation state of the familial advanced sleep phase (FASP) site, subscripts β and $p_{\text{β}}$ correspond to the phosphorylation state of the β-transducin repeat–containing protein (β-TrCP) binding site, y(t) is the concentration of casein kinases 1 ε and δ (CK1ε and CK1δ), and $u_{\text{β}}\left(t\right)$ is the concentration of the SCF^β−TrCP^ ubiquitin ligase complex, not binding to its target site. g(t)=g(t+T) where T is a period of the PER2 production.

Throughout all the PRR7 and PER2 simulations, we excluded biologically-infeasible results defined under (Equation 6), (Equation 7), (Equation 8), (Equation 9), (Equation 10) and (Equation 19), (Equation 20), (Equation 21), (Equation 22), (Equation 23), (Equation 24), (Equation 25), (Equation 26). For comparison with S values and protein synthetic costs from uniformly-sampled parameter sets, we performed the parameter optimization to maximize S or minimize the cost from each version of the models. The Nelder–Mead method in SciPy v1.3.1 (scipy.optimize) was applied to the parameter optimization.

#### Analysis of *Arabidopsis* and mouse-liver proteome data

We obtained a list of oscillating *Arabidopsis* proteins from Tables S3–S6 in Choudhary et al. (2016), based on top hit proteins of rhythmic protein spots. A comprehensive set of detected *Arabidopsis* proteins was identified by combining Data S2 in Mergner et al., 2020) and Tables S3–S6 in Choudhary et al. (2016). Among them, we identified phosphoproteins with the existing experimental evidence from *PhosPhAt* 4.0 (Durek et al., 2010).

In the case of mouse liver proteins, we followed the aforementioned procedures to collect the lists of oscillating proteins and all detected proteins, the relative abundance profiles of the oscillating proteins, and their absolute abundance levels (given the limited availability of data, we used time-snapshot data of the absolute protein abundances in mouse hepatocyte (Azimifar et al., 2014) as a rough proxy for ${<x\left(t\right)>}_{t}$ in the calculation of $c_{\text{g}}$). The list of mouse phosphoproteins was obtained from File S3 in Vlastaridis et al., 2017. We calculated $c_{\text{g}}$ of each oscillating mouse-liver protein, as detailed above.

The analysis of the mouse liver data revealed that oscillating phosphoproteins have the median abundance of $1.7\times 10^{5}$ copy number per cell (MAD = $1.6\times 10^{5}$ copy number per cell), whereas oscillating non-phospho-proteins have the median abundance of $1.1\times 10^{5}$ copy number per cell (MAD = $9.5\times 10^{4}$ copy number per cell). In other words, those phosphoproteins tend to have larger abundance levels than the non-phosphoproteins, and this result partially contributes to the enrichment of the phosphoproteins in relatively high $c_{\text{g}}$ levels (Figure 6C). Hence, we controlled for the abundance ranges of both the phospho- and non-phospho-proteins in the analysis of $c_{\text{g}}$. Specifically, we started with the observation that the probability distribution of absolute protein abundance (χ) follows a power-law $P\left(\chi \right)\propto \chi^{-\gamma }$: γ≈1 for both the phospho- and non-phospho-proteins in the range of $\chi_{\text{min}}\leq \chi \leq \chi_{\text{max}}$ where $\chi_{\text{min}}\approx 3.0\times 10^{4}$ copy number per cell and $\chi_{\text{max}}\approx 5.4\times 10^{5}$ copy number per cell. Because both the phospho- and non-phospho-proteins exhibit these power-law distributions with the almost same γ values for that χ range, the ratio of the phospho-to non-phospho-proteins remains similar for an arbitrary window of χ within the range $\chi_{\text{min}}\leq \chi \leq \chi_{\text{max}}$. In other words, as long as χ lies between $\chi_{\text{min}}$ and $\chi_{\text{max}}$, the phosphoproteins have no systematic abundance biases toward higher $c_{\text{g}}$ values. In this regard, we only chose the proteins with the abundance range of $\chi_{\text{min}}\leq \chi \leq \chi_{\text{max}}$ and obtained the fraction of phosphoproteins across the $c_{\text{g}}$ values (Figure 6C inset).

### Quantification and statistical analysis

For a parameter set with the largest S value of the degradation rate in each version of the PRR7 and PER2 models, we tested the statistical significance of the proximity of its proteosynthetic cost to the simulated minimum cost, as follows: we randomly sampled 10^6^ parameter sets of the model from physiologically-relevant ranges in Table S1, and excluded the parameter sets with the previously-defined biologically infeasible simulation results. Using the remaining parameter sets, we numerically computed the probability that the cost with a given parameter set is closer to the minimum cost (either from uniformly-sampled or optimized parameter sets) than the cost with the largest-S parameter set. The resulting probability from this one-tailed test was adopted for the *P* value of the proximity of the cost with the largest S to the minimum cost.

Spearman's ρ between S and the proteosynthetic costs in Figures 5G and 5H was calculated using scipy.stats.spearmanr in SciPy v1.3.1. To test the statistical significance of a large magnitude of the ρ value, we randomly permuted the costs against the S values, and obtained the distribution of ρ from 10^4^ sets of these randomly-paired S and costs. Using this null distribution of ρ, the one-tailed test was conducted to give the *P* value of the observed ρ.

To test the statistical significance of the enrichment of phosphoproteins in oscillating proteins in Figures 6A and 6B, we randomly selected from all detected *Arabidopsis* or mouse liver proteins the same number of proteins as the oscillating ones. For each of these 10^4^ sets of the randomly-selected proteins, we calculated the fraction of phosphoproteins. Using this null distribution of the phosphoprotein fractions, the one-tailed test was conducted to give the *P* value of the observed phosphoprotein fraction in the oscillating proteins.

We assessed the statistical significance of the positive association between $c_{\text{g}}$ and the proportions of phosphoproteins in Figure 6C and its inset, as follows: we randomly permuted $c_{\text{g}}$ values of all oscillating proteins regardless of phospho- and non-phospho-proteins, and obtained 10^4^ sets of these randomly-paired $c_{\text{g}}$ and proteins. For each of these sets, we computed the proportions of phosphoproteins across the ranges of $c_{\text{g}}$ in Figure 6C or its inset. Based on this information, we numerically computed the probability that a fold change of this randomized phosphoprotein proportion between adjacent $c_{\text{g}}$ ranges is always greater than or equal to the fold change of the observed phosphoprotein proportion, across $c_{\text{g}}$ levels in the ascending order. This probability from the one-tailed test was adopted for the *P* value of the positive association between $c_{\text{g}}$ and the phosphoprotein proportions.

For all random number generations in the above statistical significance tests, we used the Mersenne Twister in random.py of Python 3.7.4.

## Supporting citations

Bassal et al., 2020; Brennan et al., 2013; Kulak et al., 2014; Narumi et al., 2016; Pierce et al., 2009.
