# Supplementary material for: Cost-effective circadian mechanism: rhythmic degradation of circadian proteins spontaneously emerges without rhythmic post-translational regulation
Source: iScience. 2021 Jun 14;24(7):102726. doi: 10.1016/j.isci.2021.102726 (PMC8324817; doi:10.1016/j.isci.2021.102726)
Supplement: Document S1. Figures S1–S5, and Tables S1 and S2 [file mmc1.pdf]

iScience, Volume *24*

Supplemental information

Cost-effective circadian mechanism: rhythmic degradation of circadian proteins spontaneously emerges without rhythmic post-translational regulation

Roktaek Lim, Junghun Chae, David E. Somers, Cheol-Min Ghim, and Pan-Jun Kim

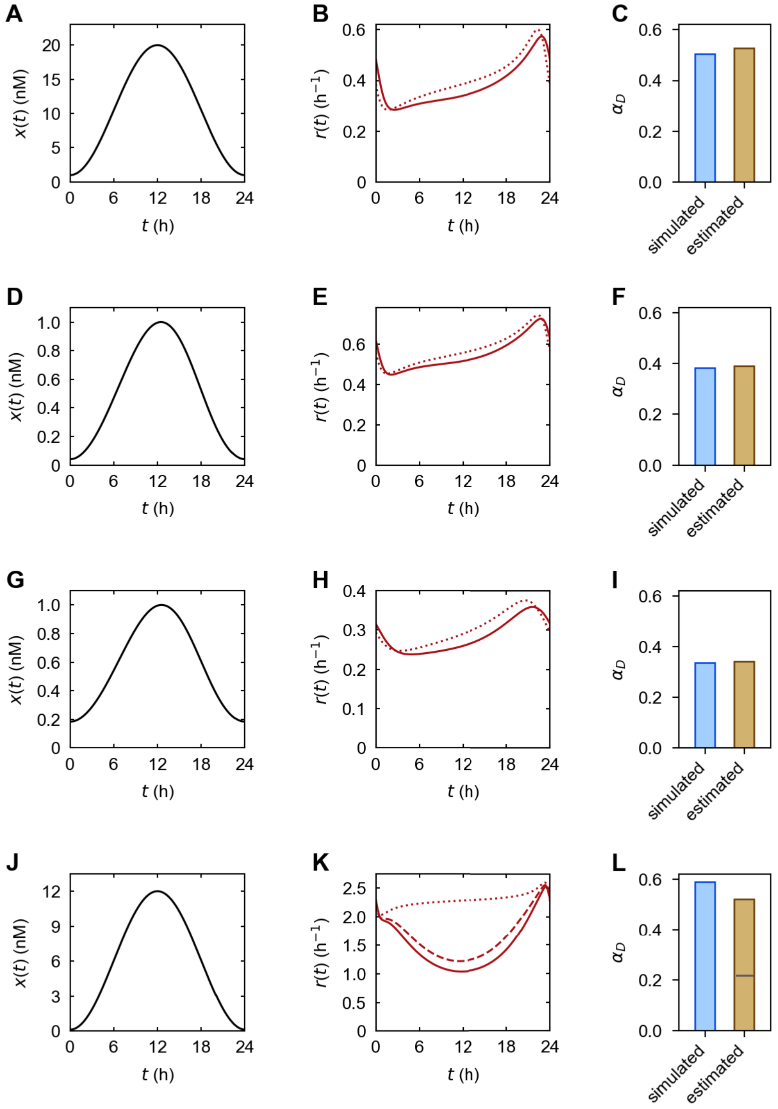

Figure S1. Comparison between simulated and mathematically estimated protein degradation rates, Related to Figure 2A. Given the protein profile $x(t)$ in (A, D, G, J), the degradation rate $r(t)$ was simulated and presented in (B, E, H, K) (solid line), respectively. The model consists of Equations 6–10 in STAR Methods, *computational modeling of protein ubiquitination without depending on other PTMs*. Model parameters in (A–I) satisfy all conditions required for the derivation of Equation 1, whereas the parameters in (J–L) satisfy the conditions required for the derivation of Equation 3, not for Equation 1 (STAR Methods, *computational modeling of protein ubiquitination without depending on other PTMs*). The simulated $r(t)$ was compared to estimates from Equation 1 (dotted line in (B, E, H, K)) or Equation 3 (dashed line in (K)). α_D of the simulated $r(t)$ in (B, E, H) was compared to estimates from Equation 1 (or, equivalently Equation 2) in (C, F, I), respectively. α_D of the simulated $r(t)$ in (K) was compared to estimates from Equation 3 in (L). The model was simulated with the following parameter values: $\bar{u} = 0.12$ nM (B), $\bar{u} = 0.16$ nM (E), $\bar{u} = 0.16$ nM (H), $\bar{u} = 0.13$ nM (K), $\bar{v} = 0$ (B, E, H, K), $a_0 = 186.8$ nM^{-1}h^{-1} (B), $a_0 = 376.9$ nM^{-1}h^{-1} (E), $a_0 = 1{,}620.8$ nM^{-1}h^{-1} (H), $a_0 = 4{,}118.7$ nM^{-1}h^{-1} (K), $a_1 = 12{,}413.9$ h^{-1} (B), $a_1 = 4{,}022.6$ h^{-1} (E), $a_1 = 13{,}400.5$ h^{-1} (H), $a_1 = 17{,}915.1$ h^{-1} (K), $a_2 = 17{,}304.3$ h^{-1} (B), $a_2 = 3{,}332.3$ h^{-1} (E), $a_2 = 6{,}964.4$ h^{-1} (H), $a_2 = 8{,}228.9$ h^{-1} (K), $r_0 = 0.71$ h^{-1} (B), $r_0 = 1.6$ h^{-1} (E), $r_0 = 0.64$ h^{-1} (H), $r_0 = 3.3$ h^{-1} (K), $q = 475.9$ h^{-1} (B), $q = 57.8$ h^{-1} (E), $q = 34.3$ h^{-1} (H), and $q = 238.7$ h^{-1} (K).

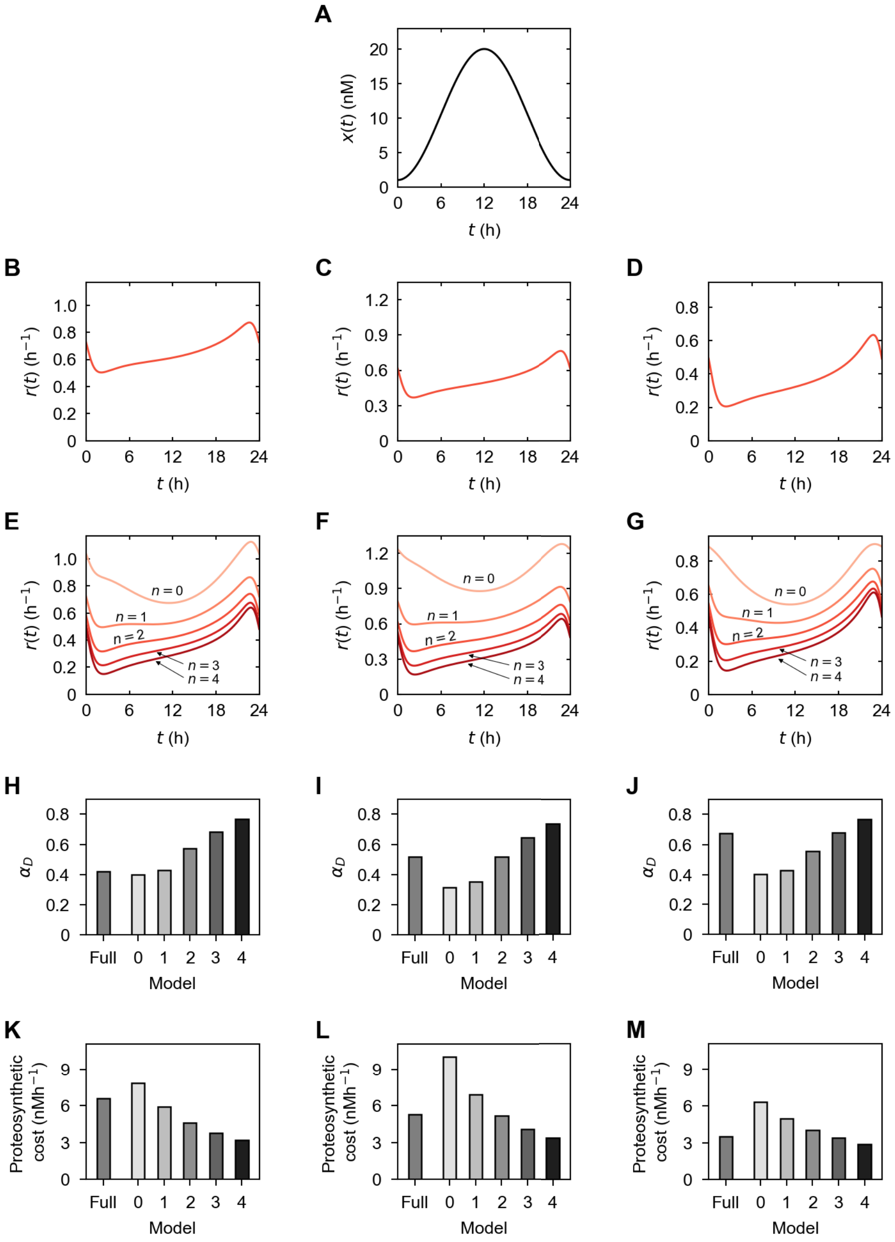

Figure S2. Comparison between simulation of multiple degradation pathways and that of individual degradation pathways, Related to STAR Methods, *model expansion for multiple degradation routes*. (A) An example of protein profile $x(t)$ for the simulation of protein degradation when more than one degradation route exists with multiple phosphorylation states of a given protein. (B–D) Given the profile $x(t)$ in (A), protein degradation rate $r(t)$ with multiple degradation routes was simulated using the equations in STAR Methods, *model expansion for multiple degradation routes*. Each simulation is based on four degradation routes associated with mono- to tetra-phosphorylation (B), three degradation routes associated with di- to tetra-phosphorylation (C), or two degradation routes associated with tri- and tetra-phosphorylation (D). (E–G) Given the profile $x(t)$ in (A), $r(t)$ with each individual degradation pathway ($n = 0, 1, \cdots, 4$) was simulated using Equations 6–10 and 19–26 in STAR Methods, *computational modeling of protein ubiquitination without depending on other PTMs* and *computational modeling of phosphorylation-dependent protein ubiquitination*. Here, n is the number of phosphorylation events required for ubiquitination and $n = 0$ corresponds to phospho-independent ubiquitination. Pathways shared between (B) and (E) are assigned the same parameter values. Likewise, (C) and (F) share the parameter values, and (D) and (G) share the parameter values. (H–M) Based on the results in (B, E), α_D and a proteosynthetic cost were computed in the case of the multiple degradation pathways (marked with "Full") or the

individual degradation pathways (marked with $n = 0, 1, \cdots, 4$) as presented in (H, K). Likewise, the results in (C, F) are associated with (I, L), and those in (D, G) are associated with (J, M). We observe that $r(t)$, α_D, and the proteosynthetic cost with multiple degradation pathways in (B, E, H, K) resemble those with a single degradation pathway of $n = 1$. $r(t)$, α_D, and the cost with multiple degradation pathways in (C, F, I, L) resemble those of $n = 2$. Lastly, $r(t)$, α_D, and the cost with multiple degradation pathways in (D, G, J, M) resemble those of $n = 3$. In other words, the simulation results with multiple degradation pathways largely reflect the results of the first available degradation pathways among the multiple ones. This tendency was observed for most of our simulation cases with various $x(t)$ profiles and parameter values in Table S1. For the illustrative purpose, the following parameter values were chosen in (B–G) ($i = 1, 2, 3, 4$): $y = 130.9$ nM (B, E), $y = 147.6$ nM (C, F), $y = 77.6$ nM (D, G), $\bar{u}_i = \bar{u} = 0.12$ nM (B, E), $\bar{u}_i = \bar{u} = 0.29$ nM (C, F), $\bar{u}_i = \bar{u} = 0.42$ nM (D, G), $z = 0$ (B–G), $\bar{v}_i = \bar{v} = 0$ (B–G), $a_{i,0} = a_0 = 249.6$ nM^{-1}h^{-1} (B, E), $a_{i,0} = a_0 = 566.0$ nM^{-1}h^{-1} (C, F), $a_{i,0} = a_0 = 1{,}754.8$ nM^{-1}h^{-1} (D, G), $a_{i,1} = a_1 = 6{,}760.6$ h^{-1} (B, E), $a_{i,1} = a_1 = 11{,}475.1$ h^{-1} (C, F), $a_{i,1} = a_1 = 11{,}348.0$ h^{-1} (D, G), $a_{i,2} = a_2 = 4{,}694.5$ h^{-1} (B, E), $a_{i,2} = a_2 = 2{,}052.8$ h^{-1} (C, F), $a_{i,2} = a_2 = 468.8$ h^{-1} (D, G), $r_{i,0} = r_0 = 2.9$ h^{-1} (B, E), $r_{i,0} = r_0 = 1.4$ h^{-1} (C, F), $r_{i,0} = r_0 = 0.93$ h^{-1} (D, G), $k_i = k = 0.014$ nM^{-1}h^{-1} (B, E), $k_i = k = 0.012$ nM^{-1}h^{-1} (C, F), $k_i = k = 0.023$ nM^{-1}h^{-1} (D, G), $q_i = q = 383.4$ h^{-1} (B, E), $q_i = q = 862.4$ h^{-1} (C, F), and $q_i = q = 1{,}024.1$ h^{-1} (D, G).

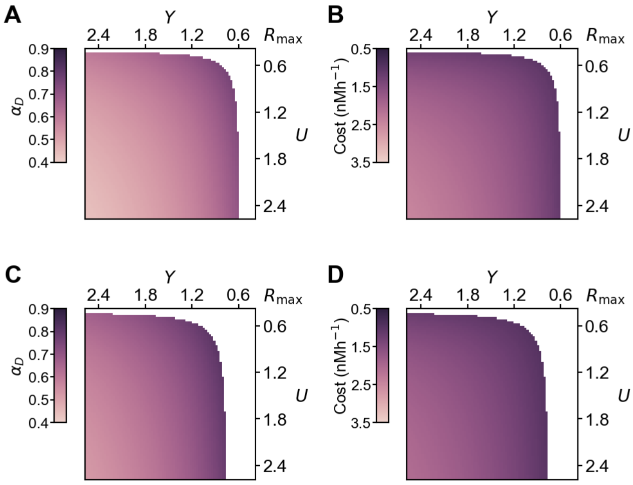

Figure S3. Effects of kinase and ubiquitin ligase activities in the case of multisite-phosphorylation-dependent ubiquitination, Related to Figure 3C. Simulations in (A–D) maintain the same protein profile $x(t)$ in Figure 3A. α_D and proteosynthetic costs are shown with varying kinase ($\propto Y$) and ubiquitin ligase ($\propto U$) concentrations, when the protein degradation depends on prior di-phosphorylation (A, B) or tri-phosphorylation (C, D). For visual guidance, Y, U, and costs are arranged in the descending order. The top-right corners of these plots correspond to $Y = U = R_{\text{max}}$. Biologically infeasible regimes are not plotted here (STAR Methods, *computational modeling of phosphorylation-dependent protein ubiquitination*). The trends of α_D and costs in (A–D) were observed for a wide range of $x(t)$ profiles and parameter values in Table S1 when $z = \bar{v} = 0$. For notations here, refer to STAR Methods, *computational modeling of protein ubiquitination without depending on other PTMs* and *computational modeling of phosphorylation-dependent protein ubiquitination*. The model in (A–D) consists of Equations 8–10 and 16–20 in STAR Methods, *computational modeling of protein ubiquitination without depending on other PTMs* and *computational modeling of phosphorylation-dependent protein ubiquitination* with the same parameter settings as Figure 3A.

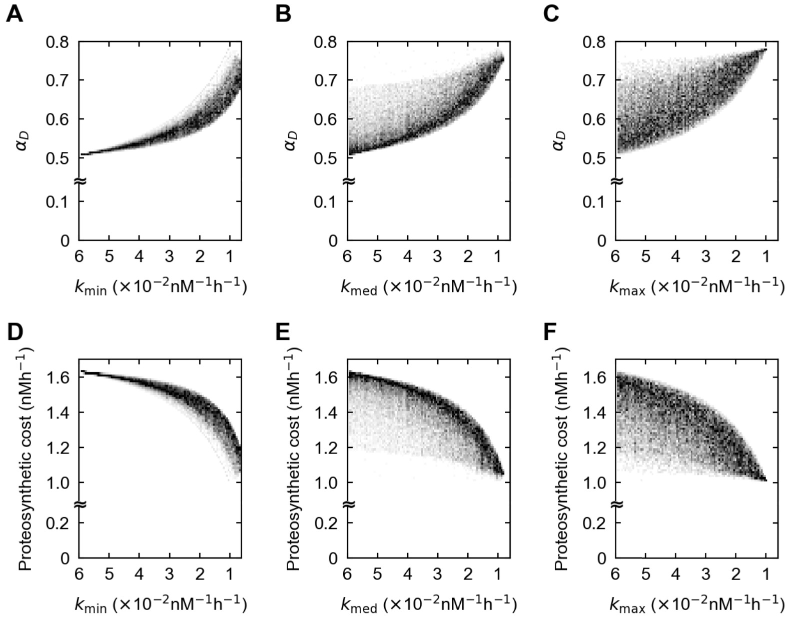

Figure S4. Effects of the lowest to highest kinase binding rates across multiple phosphosites, Related to Figure 3D. This simulation is based on protein ubiquitination dependent on prior tri-phosphorylation. k_{min}, k_{med}, and k_{max} denote the lowest, median, and highest kinase binding rates across the three phosphosites, respectively. The simulation conditions are the same as Figure 3D. For visual guidance, k_{min}, k_{med}, and k_{max} on the horizontal axes are arranged in the descending order. Density plots in (A–F) show that α_D and proteosynthetic costs are less dispersed with regards to k_{min} than to k_{med} and k_{max}. In other words, k_{min} serves as the most determining factor of α_D and cost ranges among all three binding rates.

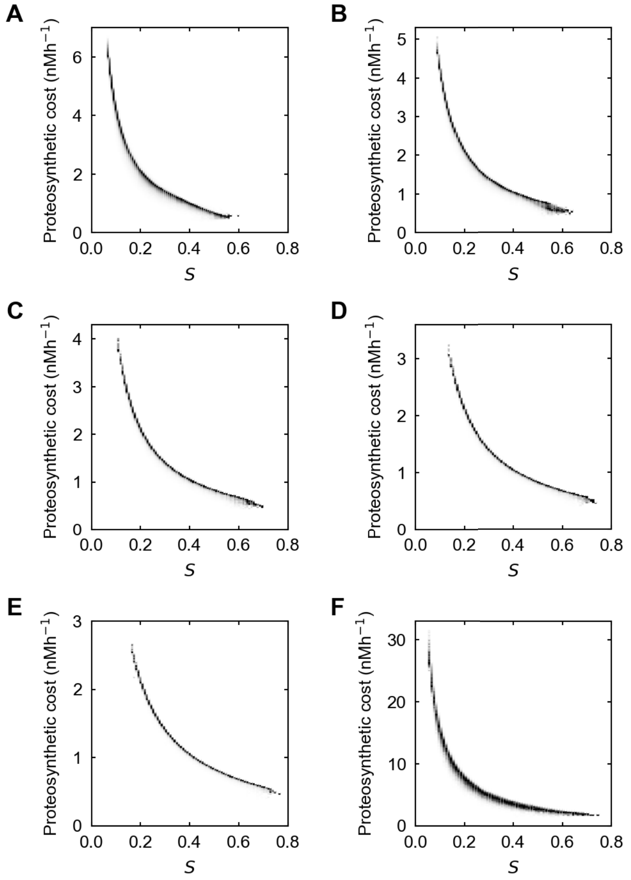

Figure S5. Simulation of alternative versions of PRR7 and PER2 degradation models, Related to Figures 5G and 5H. A density plot of S and proteosynthetic costs was obtained from each version of PRR7 and PER2 degradation models, different from the versions used in Figure 5. For these density plots, we uniformly sampled the parameter sets from physiologically-relevant ranges in Table S1. Densities were normalized to the highest density for each range of S. Resultantly, all these density plots show strong inverse correlations between S and proteosynthetic costs (Spearman's $\rho < -0.99$ and $P < 10^{-4}$; STAR Methods, *quantification and statistical analysis*). These trends are similar to the model results in Figures 5G and 5H. In the following description, n denotes the number of phosphorylation events required for ubiquitination and $n = 0$ corresponds to phospho-independent ubiquitination: (A–E) PRR7 degradation models with phosphatase and deubiquitinating enzyme activities, based on Equations 6–10 and 19–26 in STAR Methods, *computational modeling of protein ubiquitination without depending on other PTMs* and *computational modeling of phosphorylation-dependent protein ubiquitination*. $n = 0$ (A), $n = 1$ (B), $n = 2$ (C), $n = 3$ (D), or $n = 4$ (E). In the case $n > 1$, k_1, k_2, ⋯, k_n in Equations 22–25 (STAR Methods, *computational modeling of phosphorylation-dependent protein ubiquitination*) were allowed to differ from each other. (F) A full realistic PER2 degradation model in STAR Methods, *PRR7 and PER2 degradation modeling and analysis*.

Table S1

Model parameter ranges, Related to STAR Methods. The unit of min^{-1} in this table is converted to the unit of hour^{-1} in the main text and figures. For notations here, refer to Equations 6–10, 19–26 and their associated discussions in STAR Methods, *computational modeling of protein ubiquitination without depending on other PTMs* and *computational modeling of phosphorylation-dependent protein ubiquitination*. Notations of some parameters in Equations 22–26 and in other equations in STAR Methods have additional subscripts, but their values are still chosen from the following ranges of the parameters of the analogous notations. For diurnal or circadian period T, we chose $T = 24$ hour because circadian periods in this study are close to 24 hours.

Parameter	Unit	Minimum	Maximum	Sources and remarks
a_0	$\mathrm{nM}^{-1}\mathrm{min}^{-1}$	3	80	Inferred from Pierce et al., 2009.
a_1	min^{-1}	7	300	Inferred from Pierce et al., 2009.
a_2	min^{-1}	3	300	Inferred from Pierce et al., 2009.
b_0	$\mathrm{nM}^{-1}\mathrm{min}^{-1}$	3	80	Analogous to the range of a_0.
b_1	min^{-1}	3	300	Analogous to the range of a_2.
q	min^{-1}	0.5	20	Inferred from Pierce et al., 2009.
s	min^{-1}	0.5	20	Analogous to the range of q.
r_0	hour^{-1}	$\max_t\left[-\frac{x'(t)}{x(t)}\right]$ [hour^{-1}]	5	The lower limit of r_0 is based on the relation $R(\tau) \leq D(\tau) \leq 1$ in STAR Methods, *computational modeling of protein ubiquitination without depending on other PTMs*, which leads to $r_0 R(\tau) = -\frac{x'(t)}{x(t)} \leq r_0$ and then $\max_t\left[-\frac{x'(t)}{x(t)}\right] \leq r_0$. The upper limit of r_0 is roughly based on the order of magnitude of very high protein degradation rates.
k	$\mathrm{nM}^{-1}\mathrm{min}^{-1}$	0.0001	0.001	Inferred from Brennan et al., 2013.
l	$\mathrm{nM}^{-1}\mathrm{min}^{-1}$	0.0001	0.001	Analogous to the range of k.
$\max_t[x(t)]$	nM	1	20	Based on the rough range of clock protein concentrations at peak phases, as in PRR7 and PER2 cases. In the case of PRR7, we adopted 3 nM for the model simulation, but the use of 1~10 nM in general did not much alter our results. This 1~10 nM range is estimated from Bassal et al., 2020 and Nakamichi et al., 2010. In the case of PER2, we adopted 14 nM for the model simulation, based on Narumi et al., 2016.
$\bar{u}$	nM	0.1	1	Inferred from Kulak et al., 2014.
y	nM	30	300	Inferred from Narumi et al., 2016.
$\bar{v}$	nM	0	1	Analogous to $\bar{u}$ for the upper limit.
z	nM	0	300	Analogous to y for the upper limit.

Table S2

Notable S values and proteosynthetic costs from PRR7 and PER2 simulation, Related to Figures 5E and 5F. Listed are some notable values of similarity S between simulated and empirical profiles of PRR7 or PER2 degradation rates. In addition, some simulated costs of the PRR7 or PER2 synthesis are listed. The simulation was based on Equations 6–10 and 19–26, as detailed in STAR Methods, *PRR7 and PER2 degradation modeling and analysis*. Here, n denotes the model of protein degradation depending on n phosphorylation events ($n = 0$ corresponds to the model of phospho-independent degradation). S^* and S^{**} are the largest S values achieved by uniform parameter sampling and parameter optimization, respectively. c^* and c^{**} are the costs of protein synthesis from the parameter sets of S^* and S^{**}, respectively. $c^\dagger$ and $c^{\dagger\dagger}$ are the lowest costs achieved by uniform parameter sampling and parameter optimization, respectively. For S^* and $c^\dagger$, we uniformly sampled 10^6 parameter sets in physiologically-relevant ranges in Table S1. c^*, $c^\dagger$, c^{**}, and $c^{\dagger\dagger}$ take the unit of $\mathrm{nM \cdot h^{-1}}$.

**PRR7**						
n	S^*	c^*	$c^\dagger$	S^{**}	c^{**}	$c^{\dagger\dagger}$
0	0.61	0.56	0.51	0.62	0.55	0.48
1	0.70	0.50	0.48	0.72	0.47	0.45
2	0.74	0.50	0.46	0.76	0.46	0.42
3	0.76	0.45	0.43	0.78	0.45	0.42
4	0.78	0.47	0.42	0.79	0.45	0.41
**PER2**						
n	S^*	c^*	$c^\dagger$	S^{**}	c^{**}	$c^{\dagger\dagger}$
0	0.64	2.17	1.91	0.66	2.12	1.77
1	0.73	1.77	1.67	0.75	1.74	1.59
2	0.72	1.91	1.76	0.74	1.80	1.61
3	0.75	1.74	1.71	0.77	1.68	1.57
4	0.75	1.83	1.72	0.76	1.78	1.53
